# Inhaled Sedation in Patients with COVID-19-Related Acute Respiratory Distress Syndrome: An International Retrospective Study †

**DOI:** 10.3390/jcm12010012

**Published:** 2022-12-20

**Authors:** Randy Coupet, Martin Schläpfer, Thomas A. Neff, Pierre Boucher, Pierre Bailly, Martin Bellgardt, Rafael Badenes, Jose Carbonell, Tobias Becher, Caroline Varillon, Dominique Morand, Raiko Blondonnet, Jean-Michel Constantin, Bruno Pereira, Brian O’Gara, Matthieu Jabaudon

**Affiliations:** 1 Department of Anesthesiology, Critical Care and Perioperative Medicine, CHU Clermont-Ferrand, 63000 Clermont-Ferrand, France; 2 Institute of Anesthesiology, University Hospital Zurich, 8091 Zurich, Switzerland; 3 Institute of Physiology, University Zurich, 8057 Zurich, Switzerland; 4 Department of Anesthesiology and Intensive Care Medicine, Cantonal Hospital Muensterlingen, 8596 Muensterlingen, Switzerland; 5 Department of Anesthesiology and Critical Care, Ramsay Santé Private Hospital de la Loire, 42100 Saint-Etienne, France; 6 Department of Medical Intensive Care, CHRU Brest, 29609 Brest, France; 7 St. Josef-Hospital, University Hospital of Ruhr-University of Bochum, 44791 Bochum, Germany; 8 Department of Anesthesiology and Intensive Care, Hospital Clìnico Universitario de Valencia, University of Valencia, 46010 Valencia, Spain; 9 Department of Anesthesiology and Intensive Care Medicine, University Medical Center Schleswig-Holstein, Campus Kiel, 24105 Kiel, Germany; 10 Department of Medical Intensive Care, Dunkerque Hospital, 59240 Dunkerque, France; 11 iGReD—Genetics Reproduction and Development Research Unit, National Center for Scientific Research (CNRS), National Institute of Health and Medical Research (INSERM), Clermont Auvergne University, 63001 Clermont-Ferrand, France; 12 Department of Anesthesiology and Critical Care, GRC 29, DMU DREAM, Pitié-Salpêtrière Hospital, Sorbonne University, Assistance Publique-Hôpitaux de Paris, 75013 Paris, France; 13 Biostatistics and Data Management Unit, Department of Clinical Research and Innovation (DRCI), CHU Clermont-Ferrand, 63000 Clermont-Ferrand, France; 14 Department of Anesthesia, Critical Care & Pain Medicine, Beth Israel Deaconess Medical Center, Harvard Medical School, Boston, MA 02115, USA

**Keywords:** coronavirus disease 2019, acute respiratory distress syndrome, inhaled sedation, sevoflurane, isoflurane

## Abstract

Background and objectives: The coronavirus disease 2019 (COVID-19) pandemic and the shortage of intravenous sedatives has led to renewed interest in inhaled sedation for patients with acute respiratory distress syndrome (ARDS). We hypothesized that inhaled sedation would be associated with improved clinical outcomes in COVID-19 ARDS patients. Methods: Retrospective international study including mechanically ventilated patients with COVID-19 ARDS who required sedation and were admitted to 10 European and US intensive care units. The primary endpoint of ventilator-free days through day 28 was analyzed using zero-inflated negative binomial regression, before and after adjustment for site, clinically relevant covariates determined according to the univariate results, and propensity score matching. Results: A total of 196 patients were enrolled, 78 of whom died within 28 days. The number of ventilator-free days through day 28 did not differ significantly between the patients who received inhaled sedation for at least 24 h (*n* = 111) and those who received intravenous sedation only (*n* = 85), with medians of 0 (interquartile range [IQR] 0–8) and 0 (IQR 0–17), respectively (odds ratio for having zero ventilator-free days through day 28, 1.63, 95% confidence interval [CI], 0.91–2.92, *p* = 0.10). The incidence rate ratio for the number of ventilator-free days through day 28 if not 0 was 1.13 (95% CI, 0.84–1.52, *p* = 0.40). Similar results were found after multivariable adjustment and propensity matching. Conclusion: The use of inhaled sedation in COVID-19 ARDS was not associated with the number of ventilator-free days through day 28.

## 1. Key Points Summary

- Question: Compared to a standard care strategy of intravenous sedation, does inhaled sedation affect clinical outcomes in patients with COVID-19 ARDS?
- Findings: The number of ventilator-free days through day 28 of inclusion did not differ significantly between patients who received inhaled sedation for at least 24 h and those who received intravenous sedation only.
- Meaning: In this retrospective multicenter cohort of 196 patients with COVID-19 ARDS, the use of inhaled sedation with sevoflurane or isoflurane was not associated with improved clinical outcomes; however, this strategy was feasible and safe, while reducing requirements for other sedative agents.

## 2. Introduction

The surge in severe coronavirus disease 2019 (COVID-19) cases has led to an overwhelmed hospital and intensive care unit (ICU) capacity [1,2,3,4] and an international shortage of drugs [5]. Patients with COVID-19 requiring mechanical ventilation often need prolonged and high-dose sedation with hypnotics, opioids, and neuromuscular blocking agents to achieve sufficient comfort and to manage patient–ventilator dyssynchrony compared to patients with acute respiratory distress syndrome (ARDS) from other causes [6,7,8,9,10]. Ideally, sedation should be integrated into the ABCDEF bundle for ICU liberation: assessment and management of pain, both awakening and breathing trials, choosing the optimal sedative and titrating to the lightest sedation level possible, delirium assessment and management, early mobilization, and family engagement [11].

Inhaled sedation with isoflurane or sevoflurane can be delivered to ICU patients through miniaturized vaporizers added to the respiratory circuit [12]. Volatile anesthetics have been associated with better arterial oxygenation, less pulmonary edema, and decreased inflammation in preclinical models of acute lung injury [13,14,15,16,17,18]. A pilot single-center trial in ARDS patients found beneficial effects from sevoflurane compared to intravenous midazolam on gas exchange, lung epithelial injury, and inflammation [19]. However, these studies focused on lung injury induced primarily by bacteria, bacterial components, or sterile inflammation, not virus-induced ARDS. Three multicenter randomized controlled trials are currently evaluating the effects of volatile anesthetics on clinical outcomes in ARDS, including in patients with COVID-19 (ClinicalTrials.gov identifiers: NCT04415060, NCT04235608, and NCT04355962).

We conducted the international multicenter retrospective “Inhaled Sedation for COVID-19-related ARDS” (ISCA) study to investigate whether inhaled sedation would be associated with more ventilator-free days than intravenous sedation in ICU adults with severe COVID-19.

## 3. Materials and Methods

Additional details are provided in the Supplementary Materials.

### 3.1. Study Design, Population, and Data Collection

This retrospective, observational, multicenter study was conducted at 10 university and non-university hospitals in France, Germany, Spain, Switzerland, and the United States (Supplementary Table S1). This study was registered on ClinicalTrials.gov (identifier, NCT04383730) on 12 May 2020 and performed in accordance with the STrengthening the Reporting of OBservational studies in Epidemiology (STROBE) statement (Supplementary Digital Content 1) [20].

Consecutive adult patients admitted for severe COVID-19 and requiring invasive mechanical ventilation and sedation between March 2020 and May 2021 were included. There were no exclusion criteria. Sedation practices were those routinely used in the participating centers. The patients were divided into two groups: those who received inhaled sedation for ≥24 h and those who received only intravenous sedation within 28 days of enrollment.

De-identified, clinico-biological data routinely recorded in the patient health record were retrospectively collected. The patients were followed up to 28 days after enrollment.

### 3.2. Ethics and Consent

The study protocol (Supplementary Digital Content 2) was approved by ethics committees from France (IRB00010254-2020-050), Germany (D471/20), Spain (SAS/3470/2009), Switzerland (2020-01448), and the United States (2020P000326). Signed informed consent was waived by ethics committees.

### 3.3. Endpoints

#### 3.3.1. Primary Endpoint

The primary outcome was the number of ventilator-free days through day 28. The patients who died within 28 days were assigned zero ventilator-free days. A period of assisted breathing <24 h or for surgical purposes was not considered in the calculation of ventilator-free days.

#### 3.3.2. Secondary Endpoints

The secondary outcome measures were: all-cause mortality at day 28; the number of ICU-free days through day 28, durations of invasive mechanical ventilation and of controlled mechanical ventilation through day 28; physiological measures of lung function and ventilator settings on days 0–7; the duration of vasopressor support and continuous neuromuscular blockade and the need for and duration of renal replacement therapy through day 28; prone positioning and adjuvant interventions for severe ARDS through day 7; and the type, duration, and modalities of sedation practices (including measures from the ABCDEF bundle) [11] on days 0–7 and through day 28.

### 3.4. Statistical Analysis

Analyses were performed with Stata v15 (StataCorp) and R: A language and environment for statistical computing (v3.6.3) (R Foundation for Statistical Computing, Vienna, Austria) [21]. Statistical significance was established by a *p*-value of <0.05 using two-sided hypothesis tests. We did not correct for multiple comparisons, and results should be interpreted as exploratory. No missing data were imputed.

For the primary analysis of ventilator-free days through day 28, zero-inflated negative binomial regression was performed. The results were expressed with 95% confidence intervals (CI) as the odds ratio (OR) for having zero ventilator-free days through day 28 and the incidence rate ratio (IRR) for the number of ventilator-free days through day 28 when not zero. We also analyzed the primary endpoint using zero-inflated negative binomial regression after adjustment for the site (as a random effect) and the covariates determined according to the univariate results and clinical relevance. Subgroup analyses of the primary endpoint were performed after the interaction between the treatment and the predefined subgroups was tested. Propensity score matching was performed using the predicted probability of the treatment group derived from the fitted logistic regression model regression, with the covariates identified as clinically relevant after the univariate analysis included in the propensity score model. The propensity score was used to compare the variables and outcomes in a cohort of propensity-matched patients and as a covariate in the multivariable analyses. Sensitivity analyses of the primary endpoint were also performed considering distinct durations of inhaled sedation (≥2, ≥3, ≥4, ≥5, ≥7, or ≥10 days) for the patients in the inhaled sedation group. Repeated data (such as the measures of lung function through day 7) were analyzed longitudinally using mixed models to study the fixed effects group, the time point evaluation, and their interaction considering between- and within-subject variability.

## 4. Results

### 4.1. Patient Characteristics

**Figure 1:**
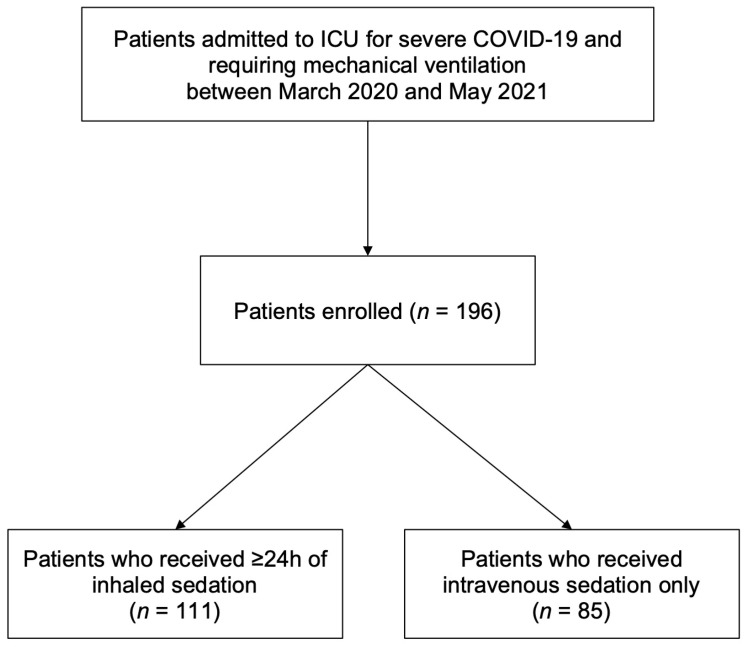
Flowchart of the study.

Among the 196 patients enrolled, 111 patients (57%) received inhaled sedation for ≥24 h through day 28, and 85 patients (43%) received intravenous sedation only (Figure 1). Past medical history, demographics and baseline (day 0) characteristics are provided in Table 1 and Table S2. The patients who received inhaled sedation were less likely to have received corticosteroids or immunosuppressant drugs in the past three months, less frequently required the use of a second sedative agent at baseline, and were more likely to receive measures from the ABCDEF bundle.

### 4.2. Outcomes

#### 4.2.1. Primary Outcome

**Figure 2:**
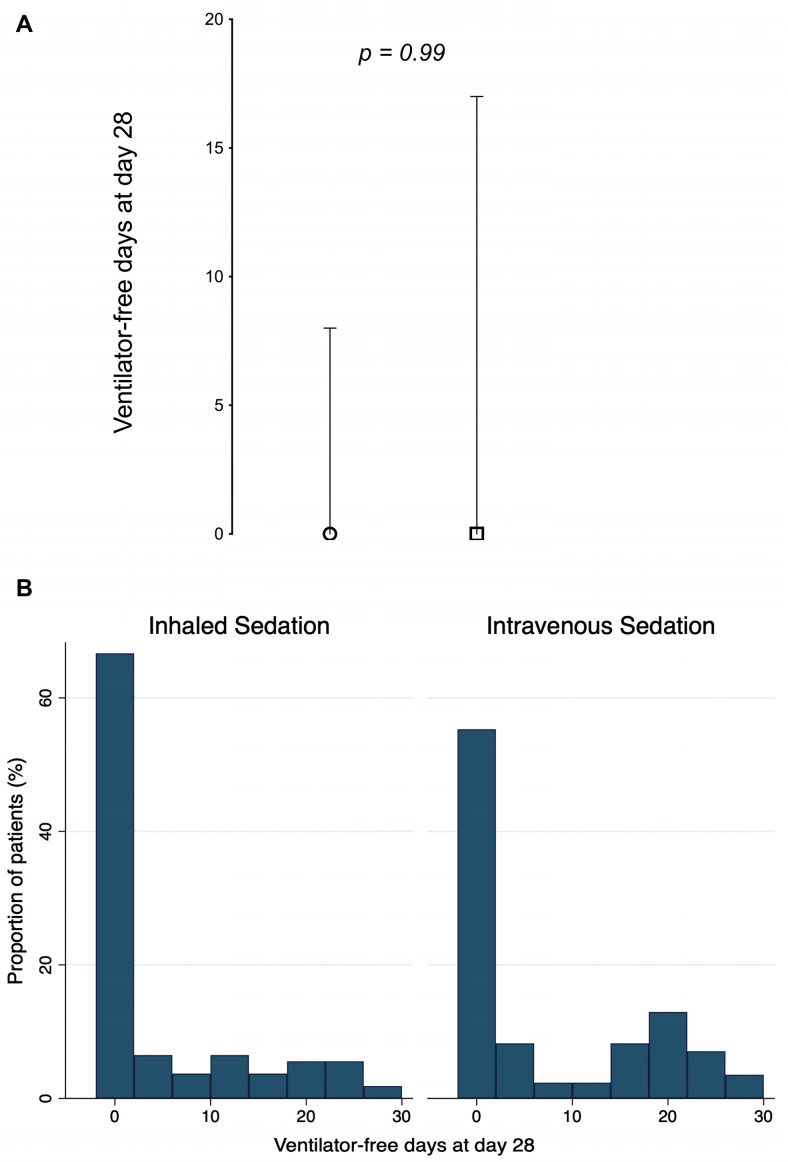
Primary outcome of ventilator-free days through day 28. (**A**) Median values (with interquartile ranges) in the patients who received inhaled sedation for at least 24 h (inhaled sedation group) and those who received intravenous sedation only (intravenous sedation group) through day 28. (**B**) Distribution of ventilator-free days through day 28 in both study groups.

The number of ventilator-free days through day 28 did not differ significantly between the inhaled sedation group (median 0, interquartile range [IQR] 0–8) and the intravenous sedation group (median 0, IQR 0–17), for an absolute difference of 0 days (95% CI, −2.91 to 2.91; *p* = 0.99) (Figure 2). A total of 71 patients (66%) receiving inhaled sedation and 46 patients (54%) receiving intravenous sedation had zero ventilator-free days (OR for having zero ventilator-free days through day 28, 1.63; 95% CI, 0.91–2.92; *p* = 0.10). In the patients with ventilator-free days through day 28 not equal to 0, the median values of ventilator-free days through day 28 were 13 days (IQR 7–21) in the inhaled sedation group and 18 days (IQR 8–20) in the intravenous sedation group (IRR 1.13; 95% CI, 0.84–1.52; *p* = 0.40) (Table 2).

The analysis of the primary endpoint provided similar results after multivariable adjustments for the site as random effects and the covariates from the univariate analysis (Table 3 and Table S3).

A propensity score and a propensity-matched cohort were developed. The variables included in the propensity score model were tidal volume, documentation of the Richmond Agitation–Sedation Scale and the confusion assessment method for the ICU by the bedside nurse, the need for a second sedative agent on day 0, and the use of specific therapies for COVID-19 on day 0 (Supplementary Table S4). There was no between-group difference in ventilator-free days through day 28 in the propensity-matched cohort (Supplementary Tables S5 and S6) or when the propensity score was used as a covariate for the multivariable analyses (Table 3).

The unadjusted sensitivity analysis showed that the risk of having zero ventilator-free days through day 28 was higher in the patients who received inhaled sedation for at least 4 days (OR for having zero ventilator-free days through day 28, 2.15; 95% CI, 1.15–4.04), 5 days (OR, 2.44; 95% CI, 1.23–4.81), or 7 days (OR, 2.36; 95% CI, 1.13–4.90) than in those who did not (Supplementary Table S7). In unadjusted subgroup analysis, the risk of having zero ventilator-free days through day 28 was higher in the patients who received inhaled sedation for more than 5 days (OR for having zero ventilator-free days through day 28, 2.44 [1.23−4.81], *p* = 0.01) (Supplementary Table S8).

#### 4.2.2. Secondary Outcomes

By day 28, 49 of the 111 patients (44%) in the inhaled sedation group and 29 of the 85 patients (34%) in the intravenous sedation group had died (unadjusted relative risk 0.85; 95% CI, 0.68–1.06; *p* = 0.14) (Table 4). The unadjusted ORs for having zero hospital-free and ICU-free days with inhaled sedation were 3.10 (95% CI, 1.36–7.10; *p* = 0.01) and 2.41 (95% CI, 1.35–4.57; *p* = 0.01), respectively, compared to intravenous sedation. There was no difference in the other secondary outcomes (duration of invasive mechanical ventilation and controlled mechanical ventilation, incidence of adverse events potentially attributable to inhaled sedation, and the need for vasopressor support or renal replacement therapy through day 28) in unadjusted analysis.

There were no between-group differences in ventilator settings, in most physiological measures of lung function or in the use of adjuvant interventions for severe ARDS through day 7 (Supplementary Table S9 and Supplementary Figures S1 and S2). However, the partial pressure of arterial carbon dioxide on day 5 and the dynamic inspiratory plateau pressure on day 2 were higher in the patients who received inhaled sedation than in those who did not (median [IQR], 49 [43; 57] vs. 44 [40; 52] mmHg, *p* for time × group interaction = 0.04 and 26 [22; 28] vs. 25 [21; 28] cmH_2_O, *p* for time × group interaction = 0.03, respectively), which was associated with the lower compliance of the respiratory system on day 6 (30 [24; 39] vs. 31 [25; 39] mL/cmH_2_O, *p* for time × group interaction = 0.01) (Supplementary Table S10).

Inhaled sedation was administered for a median duration of 5 days (IQR 3–10), and most patients received sevoflurane (Sevoflurane, Baxter International, Deerfield, IL, USA; Sevorane, AbbVie, North Chicago, IL, USA), through the Sedaconda anesthetic conserving device (Sedaconda-ACD, Sedana Medical, Danderyd, Sweden); inhaled sedation use was associated with fewer days with intravenous sedation through day 28 (Table 4).

The details on sedation practices and ABCDEF bundle use, as per the treating clinicians, through day 7 are provided in Supplementary Figures S3 and S4, Supplementary Table S11; the use of multiple sedatives was often required in patients from both groups, and the implementation rates for measures from the ABCDEF bundle were low.

## 5. Discussion

In this retrospective study of patients with COVID-19 ARDS, the use of inhaled sedation did not affect the number of ventilator-free days through day 28 compared to intravenous sedatives only.

Although potential benefits of volatile anesthetics, such as through decreased awakening and extubation times in comparison with intravenous sedatives, have been previously reported in non-COVID-19 patients [22,23], their effect on clinical outcomes remains unknown in COVID-19 patients. We found that higher durations of inhaled sedation were associated with higher odds of having zero ventilator-free days in the subgroup analysis, but this effect on ventilator-free days was not found consistently with increasing durations of inhaled sedation in the sensitivity analyses. Therefore, these results should be interpreted with caution. In our study, in which the median duration of inhaled sedation was 5 days, inhaled sedation did not affect 28-day mortality or duration of mechanical ventilation, supporting the feasibility and overall safety of its use for longer durations [24]. However, the precise effects of prolonged inhaled sedation in COVID-19 patients require further investigation, as most studies have enrolled non-COVID-19 patients and have shorter durations (≤48–72 h) [19,22,25].

Compared to those with ARDS from other causes, patients with COVID-19 ARDS commonly require higher doses of sedatives and opioids, which are, in turn, associated with prolonged coma and increased mortality [26]. Our results are in line with these findings with frequent use of multiple agents, including benzodiazepines, to reach sedation goals in both the patients receiving and not receiving inhaled sedation. In COVID-19 patients, the use of benzodiazepines for sedation and poor implementation of the ABCDEF bundle are associated with a higher risk of delirium [8]. Interestingly, the documentation of a sedation score by the bedside nurse was associated with more ventilator-free days after univariate analysis in our study (Supplementary Table S3), supporting the benefits of the bundle in both COVID-19 and non-COVID-19 patients [11,27]. The recent surge in COVID-19 cases, which has required the rapid extension of ICU capacities associated with a risk of staff, ventilator, or drug shortages, may explain why the ABCDEF bundle was not often implemented in our cohort. In association with non-protocolized mechanical ventilation, this might have influenced clinical outcomes and may explain, at least partially, the between-group differences in ICU-free and hospital-free days and the important variability in 28-day mortality rates across the study centers (Supplementary Table S1).

It is also possible that the potential lung-protective effects of volatile anesthetics found in preclinical studies and in one pilot clinical trial [14,17,18,19] might differ between COVID-19 and non-COVID-19 patients. The presence of an overwhelmed inflammatory response in COVID-19 remains controversial and might not be as obvious as initially thought [28,29,30,31,32]. In our study, inhaled sedation was not associated with improved oxygenation, in contrast to previous reports in patients without [19] and with COVID-19, [33,34,35] and our longitudinal analyses suggested a potential increase in the partial pressure of arterial carbon dioxide and dynamic inspiratory plateau pressure with inhaled sedation. However, there was no strict protocol for mechanical ventilation in our study, and time × group interactions were only significant on day 5 for carbon dioxide and on day 0 for plateau pressure, which is inconsistent with the available evidence in non-COVID-19 and COVID-19 patients [9,23,24,25,33,34,35,36].

No serious adverse effects potentially attributable to inhaled sedation with isoflurane or sevoflurane were reported in our study. There were no cases of malignant hyperthermia, and the incidence of diabetes insipidus, which has been reported in a few patients after the prolonged use of high-dose sevoflurane [37], was similar in the patients from our cohort who received inhaled sedation and those who did not. These findings confirm the overall safety of inhaled sedation for invasively ventilated patients, including those with ARDS [19,23,24,25,36]. They also support the efficacy of volatile anesthetics for ICU sedation, as recently confirmed by a large phase 3, randomized controlled non-inferiority trial of isoflurane vs. propofol in critically ill patients without COVID-19 [25]. In that trial, as in other studies, [19,22,24] volatile anesthetics were efficacious as the sole sedatives in non-COVID-19 patients and significantly reduced the requirement for other sedative and opioid agents in COVID-19 patients [9,33,34,35]. Consistent with these findings, the use of inhaled sedation was associated with shorter durations of intravenous sedation (regression coefficient: −0.43; Table 4) in our study.

Our study has several important limitations. First, our cohort was assembled retrospectively, with a risk of selection bias, as well as bias in the detection of baseline clinical features and clinical outcomes. In addition, the choice of sedative agents in this study were influenced both by clinical experience and drug availability. Because inhaled sedation use was not randomly allocated in this study, a propensity analysis was performed to adjust for confounding in treatment selection; however, this cannot completely control the effect of confounding, and only randomized trials will be able to investigate causality. Second, the choice of our primary endpoint (ventilator-free days through day 28), although standard in ICU research, may not be the most appropriate in patients with COVID-19 who may experience prolonged periods of respiratory failure. Third, we defined baseline (day 0, the date of enrollment in the study) as the date when patients were both admitted to a participating center and were receiving mechanical ventilation and sedation. However, inhaled sedation was initiated later than day 0 in some patients in our study and was not always administered on successive days, which complicates the interpretation of our results, such as those from the sensitivity analyses. Fourth, we were unable to capture opioid requirements and further to evaluate whether they could be reduced with inhaled sedation, as suggested by previous reports [9,22,33,34,35]. However, consistent with previous findings, the need for intravenous sedatives was decreased when inhaled sedation was used. In addition, the risk of developing delirium under inhaled sedation compared with intravenous sedation was not specifically analyzed in our cohort and warrants further investigation [38]. Fifth, the patients in our cohort mainly received sevoflurane through the Sedaconda-ACD (Sedana Medical, Danderyd, Sweden), and whether the choice of other volatile anesthetics and/or device used for ICU sedation could impact clinical outcome remains undetermined.

## 6. Conclusions

In this retrospective multicenter study, the use of inhaled sedation did not significantly affect the number of ventilator-free days through day 28 among mechanically ventilated adults with COVID-19 ARDS. Randomized controlled trials are warranted to assess the effects of inhaled sedation on clinical outcomes in COVID-19 ARDS.

## Figures and Tables

**Table 1:** Baseline demographics, clinical and biological data.

Variable	No. of Available Individuals	Whole Cohort	Inhaled Sedation Group	IV Sedation Group	*p*-Value *
		(*n* = 196)	(*n* = 111)	(*n* = 85)	
***Demographics***					
Age, mean ± SD	196	66 ± 9	67 ± 10	66 ± 9	0.37
Men, *n* (%)	194	150 (77)	84 (76)	66 (79)	0.71
Body mass index, median (IQR), kg/m^2^	193	28.1 [24.8; 31.8]	28.7 [24.8; 31.9]	27.1 [24.7; 31.8]	0.85
***Type of structure of admission, n (%)***					
Intensive care unit	193	189 (98)	107 (97)	82 (99)	0.67
Step-down unit		2 (1)	1 (1)	1 (1)	
Operating room		1 (1)	1 (1)	0 (0)	
Intermediate care unit		1 (1)	1 (1)	0 (0)	
***Clinical data***					
Temperature (°C)	189	37.0 [36.5; 37.7]	37.1 [36.5; 37.8]	37.0 [36.5; 37.6]	0.58
Heart rate (/min)	193	80 [66; 99]	80 [66; 100]	80 [67; 98]	0.99
Mean blood pressure (mmHg)	190	75 [67; 88]	75 [66; 87]	75 [68; 88]	0.82
Supraventricular tachycardia	191	10 (5)	5 (5)	5 (6)	0.64
Atrial fibrillation	187	8 (4)	5 (5)	3 (4)	0.76
***Treatments***					
Receiving vasopressors, *n* (%)	192	152 (79)	86 (79)	66 (80)	0.92
Renal replacement therapy, *n* (%)	195	9 (5)	5 (5)	4 (5)	0.93
Receiving antibiotics, *n* (%)	195	162 (83)	89 (81)	73 (86)	0.36
SARS-CoV-2 specific treatment, *n* (%)	194	59 (30)	27 (24)	32 (39)	0.03
*Tocilizumab*	59	2 (3)	1 (4)	1 (3)	
Steroid treatment in the last 24 h, *n* (%)	194	109 (56)	62 (57)	47 (55)	0.83
*Dexamethasone*	109	92 (84)	56 (90)	36 (77)	
***Mechanical ventilation data***					
HFNO before intubation	195	92 (47)	43 (39)	49 (58)	0.01
NIV before intubation	195	61 (31)	43 (39)	18 (21)	0.01
Ventilation mode					10^−4^
*Volume-controlled ventilation*	193	86 (45)	57 (53)	29 (34)	
*Pressure-controlled ventilation*	193	56 (29)	39 (36)	17 (20)	
*Pressure-support ventilation*	193	14 (7)	6 (6)	4 (31)	
Dynamic plateau pressure (cmH_2_O)	125	26 [22; 29]	26 [22; 28]	25 [22; 30]	0.89
PEEP (cmH_2_O)	195	12 [10; 14]	12 [10; 14]	12 [10; 14]	0.21
Expired tidal volume (mL)	193	433 [383; 484]	443 [389; 485]	422 [379; 481]	0.19
V_T_ (mL/kg PBW)	190	6.53 [5.76; 7.28]	6.64 [4.99; 7.38]	6.35 [5.55; 7.12]	0.04
Compliance of the respiratory system (mL/cmH_2_O)	125	33.8 [27.0; 41.4]	33.9 [28.1; 42.5]	33.8 [26.3; 40.0]	0.52
Driving pressure (cmH_2_O)	125	13 [10; 16]	13 [10; 15]	12 [10; 16]	0.75
Airway resistance (cmH_2_O/L/s)	67	14 [12; 17]	14 [12; 17]	15 [11; 27]	0.99
***Sedation***					
Receiving sedation	196	196 (100)	111 (100)	85 (100)	10^−4^
If intravenous sedation	196	154 (79)	70 (63)	84 (99)	10^−4^
*Sedation with propofol*	154	116 (75)	52 (74)	64 (76)	0.3
*Sedation with midazolam*	154	36 (23)	16 (23)	20 (24)	0.3
*Sedation with another agent agent*	154	2 (1)	2 (3)	0 (0)	0.3
If inhaled sedation	196	41 (15)	41 (37)	0 (0)	10^−4^
*Sedation with sevoflurane*	41	29 (71)	29 (71)	0 (0)	
*Sedation with isoflurane*	41	12 (29)	12 (29)	0 (0)	
*Use of Sedaconda-ACD-S*	41	39 (95)	39 (95)	0 (0)	
*Use of ventilator/vaporizer*	41	2 (5)	2 (5)	0 (0)	
Use of a second sedative agent	194	126 (65)	59 (54)	67 (79)	10^−4^
***Biological data***					
PaO_2_/FiO_2_ (mmHg)	184	132 [98; 170]	128 [94; 165]	135 [104; 174]	0.48
PaCO_2_ (mmHg)	192	45 [40; 52]	46 [41; 52]	44 [38; 51]	0.1
Serum lactate (mmol/L)	167	1.3 [1.1; 1.8]	1.4 [1.0; 1.8]	1.3 [1.1; 1.8]	0.56
Arterial pH	192	7.34 [7.30; 7.40]	7.34 [7.3; 7.38]	7.35 [7.26; 7.40]	0.95
Serum creatinine (µmol/L)	177	79 [61; 107]	76 [62; 106]	87 [61; 113]	0.42

* *p*-values were calculated for comparisons between patients receiving inhaled sedation (for at least 24 h) and those receiving only intravenous sedation through day 28. Percentages are expressed out of available individuals for each variable and may not sum to 100% due to rounding. Quantitative variables are expressed as median [IQR], qualitative variables as number (%), unless specified otherwise. SD: standard deviation; IQR: interquartile range; SARS-CoV-2: severe acute respiratory syndrome coronavirus 2; HFNO: high-flow nasal oxygenation; NIV: non-invasive ventilation; PEEP: positive end expiratory pressure; V_T_: tidal volume; PBW: predicted body weight; PaO_2_: partial pressure of oxygen in arterial blood; FiO_2_: inspired fraction of oxygen; PaCO_2_: partial pressure in carbon dioxide in arterial blood.

**Table 2:** Unadjusted analysis of ventilator-free days through day 28 using zero-inflated negative binomial regression.

		Univariate Analysis (Zero-Inflated Negative Binomial Regression)			
	VFD28, Median (IQR)	Patients with Zero VFD28, *n* (%)	OR (95%CI) for Having Zero VFD28, *p*-Value *	VFD28 if VFD28 Not Zero, Median (IQR)	IRR (95%CI) for the Number of VFD28 if Not Zero, *p*-Value *
**Sedation**					
Intravenous	0 [0; 17] (*n* = 85)	46 (54)	reference	18 [8; 20] (*n* = 39)	reference
Inhaled	0 [0; 8] (*n* = 108)	71 (66)	1.63 [0.91; 2.92], *p* = 0.10	13 [7; 21] (*n* = 37)	1.13 [0.84; 1.52], *p* = 0.40

* p-values were calculated for comparisons between patients receiving inhaled sedation (for at least 24 h) and those receiving only intravenous sedation through day 28. VFD28: ventilator-free days through day 28; OR: odds ratio; CI: confidence interval; IQR: interquartile range; IRR: incident rate ratio.

**Table 3:** Multivariable analyses of ventilator-free days through day 28 using zero-inflated negative binomial regression.

	Multivariable Analysis of VFD28		
	Zero-Inflated Negative Binomial Regression		
	Number of Complete Cases Available	OR [95%CI] for Having Zero VFD28, *p*-Value	IRR [95%CI] for the Number of VFD28 if Not Zero, *p*-Value
Adjusted for site (random effect)	193	1.63 [0.91; 2.92], *p* = 0.10	1.23 [0.89; 1.71], *p* = 0.22
Adjusted for site (random effect) and heterogeneity *	151	1.80 [0.85; 3.85], *p* = 0.13	1.15 [0.84; 1.57], *p* = 0.39
Adjusted for site (random effect) and confounding **	158	1.33 [0.67; 2.64], *p* = 0.42	1.24 [0.90; 1.72], *p* = 0.19
Adjusted for site (random effect), heterogeneity *, and confounding **	133	1.67 [0.70; 3.98], *p* = 0.25	1.12 [0.80; 1.55], *p* = 0.51

* Heterogeneity represents clinically relevant baseline variables associated with VFD28 after univariate analysis: age, a medical history of arterial hypertension, and the partial pressure of arterial-oxygen-to-fraction-of-inspired-oxygen ratio, the need for vasopressor support, the documentation of an agitation–sedation scale by the bedside nurse, and serum creatinine on day 0. ** Confounding represents patient selection bias due to nonrandomized assignment of treatment. Propensity score derived from a logistic equation for each patient was incorporated as a continuous variable into outcome analysis to adjust for possible confounding. VFD28: ventilator-free days through day 28; OR: odds ratio; CI: confidence interval; IQR: interquartile range; IRR: incident rate ratio.

**Table 4:** Secondary endpoints as evaluated at day 28 after inclusion.

Variable	No. of Available Individuals	Whole Cohort (*n* = 196)	Inhaled Sedation (*n* = 111)	Intravenous Sedation (*n* = 85)	*p*-Value *	Relative Risk or Regression Coefficient (95% CI)
Death at day 28, *n* (%)	196	78 (40)	49 (44)	29 (34)	0.14	0.85 (0.68; 1.06)
ICU-free days through day 28	192	0 [0; 4]	0 [0; 0]	0 [0; 12]	0.004	−0.48 (−0.81; −0.16)
Hospital-free days through day 28	189	0 [0; 0]	0 [0; 0]	0 [0; 0]	0.002	−0.38 (−0.62; −0.14)
Total duration of invasive mechanical ventilation through day 28, days	191	14 [7; 24]	15 [6; 23]	12 [7;26]	0.78	−0.03 (−0.27; 0.20)
Total duration of controlled mechanical ventilation through day 28, days	189	11 [4; 21]	11 [5; 21]	11 [4;21]	0.9	0.02 (−0.24; 0.28)
Total duration of dobutamine support, days	176	0 [0; 0]	0 [0; 0]	0 [0; 0]	0.19	0.12 (−0.06; 0.29)
Total duration of epinephrine support, days	175	0 [0; 0]	0 [0; 0]	0 [0; 0]	0.21	0.04 (−0.02; 0.11)
Total duration of dopamine support, days	174	0 [0; 0]	0 [0; 0]	0 [0; 0]	0.26	−0.01 (−0.04; 0.01)
Total duration of norepinephrine support, days	188	8.5 [4.0; 17.0]	10.0 [5.0; 17.0]	7.0 [3.0; 18.0]	0.06	0.24 (−0.01; 0.49)
Need for RRT, *n* (%)	192	70 (36)	42 (39)	28 (33)	0.37	1.19 (0.81; 1.75)
Total duration of RRT, days	70	9.5 [4.0;20.0]	10.5 [4.0;16.0]	9.5 [4.5;22.0]	0.46	−0.14 (−0.53; 0.24)
Nephrogenic diabetes insipidus, *n* (%)	144					
*Suspected*		7 (5)	1 (1)	6 (8)	0.05	0.13 (0.02; 1.04)
*Confirmed*		4 (3)	2 (3)	2 (3)	0.79	0.77 (0.11; 5.33)
Total duration of inhaled sedation, days	107	0 [0; 0]	5.0 [3.0; 10.0]	0 [0; 0]	<10^−3^	1.88 (1.72; 2.03)
Total duration of intravenous sedation, days	192	11.0 [4.0; 19.5]	8.0 [3.0; 17.0]	12.0 [7.0; 24.0]	0.001	−0.43 (−0.68; −0.18)
Total duration of neuromuscular blockade, days	187	0 [0; 4]	1 [0; 5]	0 [0; 3]	0.19	0.18 (−0.09; 0.46)

Durations are expressed in days since day 0 (study entry). Quantitative variables are expressed as median [interquartile range] and qualitative variables as number (%), unless specified otherwise. * *p*-values were calculated for comparisons between patients receiving inhaled sedation (for at least 24 h) and those receiving only intravenous sedation through day 28. Effect sizes are expressed as relative risks for categorical variables and regression coefficients for quantitative variables (as expressed for each one-log change in value), with 95% confidence intervals (CI). ICU: intensive care unit; RRT: renal replacement therapy; NMB: neuromuscular blockade.

## Data Availability

All data generated or analyzed during this study are included in this published article and its additional files.

## Supplementary Materials

The following supporting information can be downloaded at: https://www.mdpi.com/article/10.3390/jcm12010012/s1, Figure S1. Modes of invasive mechanical ventilation on study days 0–7 in the patients who received inhaled sedation for at least 24 h (inhaled sedation group) and those who received intravenous sedation only (intravenous sedation group) through day 28. Figure S2. Adjunctive therapies for acute respiratory distress syndrome used on study days 0–7 in the patients who received inhaled sedation for at least 24 h (inhaled sedation group) and those who received intravenous sedation only (intravenous sedation group) through day 28. Figure S3. Agents of sedation used on study days 0–7 in the patients who received inhaled sedation for at least 24 h (inhaled sedation group) and those who received intravenous sedation only (intravenous sedation group) through day 28. Figure S4. Components of the ABCDEF bundle implemented on study days 0–7 in the patients who received inhaled sedation for at least 24 h (inhaled sedation group) and those who received intravenous sedation only (intravenous sedation group) through day 28. Table S1. List of participating centers, number of patients included per center, and number of patients who died by day 28 in each center. Table S2. Demographics and medical history. Table S3. Univariate analysis of the primary endpoint using demographic variables, variables from past medical history, variables from the day before inclusion, and variables from the day of inclusion. Table S4. Baseline variables integrated in the propensity score that was used to predict probability of receiving inhaled sedation or not, and derived from the fitted regression model (logistic regression). Table S5. Selected characteristics (demographics, medical history, data from study day 0) according to treatment assignment (inhaled versus intravenous sedation) in propensity-matched patients. Table S6. Primary and secondary endpoints according to treatment assignment (inhaled versus intravenous sedation) in propensity-matched patients. Table S7. Sensitivity analysis of the primary endpoint (ventilator-free days through day 28) when defining groups based on the length of inhaled sedation received (≥2, ≥3, ≥4, ≥5, ≥7 or ≥10 days). Table S8. Subgroup unadjusted analysis of the primary endpoint (ventilator-free days through day 28). Table S9. Mechanical ventilation data and adjunctive ARDS therapies received during the first seven days after inclusion. Table S10. Physiological measures of lung function and ventilator settings through day 7 in patients receiving inhaled sedation and those receiving intravenous sedation only. Table S11. Sedation practices and measures from the ABCDEF bundle implemented during the first seven days after inclusion. References cited in Supplementary Materials [39,40].

## Glossary

ARDS	acute respiratory distress syndrome
CI	confidence interval
COVID-19	coronavirus disease 2019
ICU	intensive care unit
IQR	interquartile range
IRR	incidence rate ratio
ISCA	inhaled sedation for COVID-19-related ARDS
OR	odds ratio
STROBE	STrengthening the Reporting of OBservational studies in Epidemiology
