# Supplementary material for: Inhaled Sedation in Patients with COVID-19-Related Acute Respiratory Distress Syndrome: An International Retrospective Study
Source: J Clin Med. 2022 Dec 20;12(1):12. doi: 10.3390/jcm12010012 (PMC9821008; doi:10.3390/jcm12010012)
Supplement: Supplementary file 1 [file jcm-12-00012-s001.zip › Supplementary Digital Content 2_Study_Protocol_v2_ISCA.pdf]

Supplementary Digital Content 2 - Study Protocol

PROTOCOLE D'ETUDE NON INTERVENTIONNELLE
RECHERCHE "SUR DONNEES"

Sédation inhalée dans la prise en charge des patients de réanimation avec syndrome de détresse respiratoire aiguë: recherche sur données médicales en période de pandémie à Coronavirus SARS-CoV2 (COVID-19)

PROTOCOL FOR A NON-INTERVENTIONAL STUDY
WITH DATA COLLECTED FROM THE PATIENT'S HEALTH RECORD

Inhaled Sedation in COVID-19-related Acute respiratory distress syndrome (ISCA): an international research data study in the recent context of widespread disease resulting from the 2019 (SARS-CoV2) coronavirus pandemics (COVID-19)

VERSION N°2 - 04/05/2020

N° Inserm	N°IDRCB	N° CPP/CEEI	N° MESR	N°CNIL/CCTIRS
**xxx**	**xxx**	France: IRB 00010254-2020-050 (Comité d'éthique pour la Recherche en Anesthésie-Réanimation - CERAR, SFAR)<br>Germany: approval D471/20 (Ethikkommission der medizinischen Fakultät der Christian-Albrechts-Universität zu Kiel)<br>Spain: approval SAS/3470/2009 (Comité de Ética de la Investigación con Medicamentos del Hospital Clínico Universitario de Valencia)	**xxx**	Méthodologie n°4

Study sponsor:

C.H.U. de Clermont-Ferrand
58 Rue de Montalembert
63003 Clermont-Ferrand Cedex 1, France

Study methodology:

Bruno PEREIRA, PhD Biostatistics
Biostatistical Unit, Department of Clinical Research and Innovation (DRCI)
CHU Clermont-Ferrand
58, Rue Montalembert
63003 Clermont-Ferrand Cedex 1, France
Email: bpereira@chu-clermontferrand.fr

Coordinating Investigator:
Dr JABAUDON Matthieu, MD PhD
Pôle de Médecine Périopératoire, CHU Clermont-Ferrand, Equipe "Approche translationnelle des lésions épithéliales et de leur réparation"; Université Clermont Auvergne, CNRS UMR 6293, INSERM U1103, GReD, Clermont-Ferrand, France; Division of Allergy, Pulmonary and Critical Care, Vanderbilt University Medical Center, Nashville, USA
Address: 1 Place Lucie et Raymond Aubrac
63003 Clermont-Ferrand Cedex 1, France
Tel: 33626289858
Fax: 33473750500
Email: **mjabaudon@chu.clermontferrand.fr**

National investigators:
Pr CONSTANTIN Jean-Michel, MD PhD
Department of Anesthesiology and Critical Care, Pitié-Salpêtrière Hospital, Sorbonne University, GRC 29, AP-HP, DMU DREAM, Paris, France
Email: **jean-michel.constantin@aphp.fr**

Dr BECHER Tobias, MD
Department of Anesthesiology and Intensive Care Medicine, University Medical Center Schleswig-Holstein, Campus Kiel, Kiel, Germany
Email: **tobias.becher@uksh.de**

Dr BADENES Rafael, MD PhD DESA MSc
Department of Anesthesiology and Intensive Care, Hospital Clínico Universitario de Valencia, Valencia, Spain
Email: rafaelbadenes@gmail.com

Pr BECK-SCHIMMER Beatrice, MD
Institute of Physiology and Zurich Centre for Integrative Human Physiology, University of Zurich and Institute of Anaesthesiology, University Hospital Zurich, Zurich, Switzerland
Email: beatrice.beckschimmer@uzh.ch

Dr SCHLAPFER Martin, MD
Institute of Physiology and Zurich Centre for Integrative Human Physiology, University of Zurich and Institute of Anaesthesiology, University Hospital Zurich, Zurich, Switzerland
Email: martin.schlaepfer@usz.ch

Dr O'GARA Brian P.
Department of Anesthesia, Critical Care & Pain Medicine, Beth Israel Deaconess Medical Center, Boston, USA
Email: bpogara@bidmc.harvard.edu

SUMMARY

IMPORTANT NOTICE 4

Proposal's context, positioning and objective(s) 4

Context 4

Research hypothesis and objectives 6

Study population 6

Description 6

Inclusion Criteria 7

Non-inclusion Criteria 7

Recruitment procedure 7

Study design and methods 7

Data collection 7

Primary outcome measure 8

Secondary outcome measures 8

Origin and nature of the personal data collected 9

Data flow and management 10

Data access 10

Data source 11

Data privacy 11

Provisional study timeline 11

Summary table of patient follow-up 12

Statistics and data analysis 12

Sample size estimation 12

Data analysis 13

General aspects 13

Primary analysis 13

Secondary analysis 14

Method for missing data 15

Missing primary outcome data 15

Missing secondary outcome data 15

Subgroups 15

Study costs and funding 16

Risk management strategy 16

Rules for publication and authorship 16

List of participating centers - (Under construction) - 17

References 19

☐ Single-center study

☐ French multicenter study

☐ European multicenter study

☐ **International multicenter study**

IMPORTANT NOTICE

This study protocol has been designed in the recent context of widespread disease resulting from the 2019 (SARS-CoV2) coronavirus pandemics (COVID-19) (www.who.int/emergencies/diseases/novel-coronavirus-2019) in order to provide rapid, yet scientifically rigorous, evaluation of the practice of inhaled sedation with halogenated agents (isoflurane or sevoflurane) in patients admitted to intensive care units that already use this technique as current sedation practice.

The main objective of the non-interventional ISCA study is to assess whether the use of inhaled sedation might be associated with improved clinical outcomes in patients with COVID-19-related acute respiratory distress syndrome.

Proposal's context, positioning and objective(s)

Context

The acute respiratory distress syndrome (ARDS) is the most severe and lethal complication of COVID-19, and healthcare resource utilizations are currently being heavily challenged in most countries worldwide, with a high risk that some intensive care resources, such as the number of ventilators to allow management all patients, may be insufficient to face the current surge in ARDS cases[1–4]. There is therefore an urgent need to evaluate candidate therapies that may impact clinical outcomes in patients with COVID-19-related ARDS[5,6] and potentially be relevant to current public health issues, in accordance with the international efforts by the World Health Organization (WHO) (Global research on coronavirus disease) and most international public health organizations.

Beyond the current efforts to find specific antiviral therapies or vaccines, improving supportive care and treatment options for patients with COVID-19-related ARDS, in accordance with up-to-date guidelines on the management of critically ill patients with COVID-19 (Surviving Sepsis Campaign: Guidelines on the Management of Critically Ill Adults with Coronavirus Disease 2019; The Australian and New Zealand Intensive Care Society (ANZICS) COVID-19 Guidelines; Recommandations d'experts SRLF-SFAR-SFMU-GFRUP-SPILF sur la prise en charge en réanimation des

patients en période d'épidémie à SARS-CoV2), is of major importance[1,7,8]. Indeed, given the number of intensive care unit (ICU) patients for whom the question of sedation applies during the current COVID-19 outbreak[9–11], any sedation practice that would be associated with improved clinical outcomes could have significant economic and public health implications.

In this perspective, the rationale supporting inhaled sedation with halogenated agents (such as isoflurane or sevoflurane) as a way to improve lung function, to decrease the inflammatory response, and to possibly improve patient outcome is strong.

Numerous clinical trials support the efficacy and safety of volatile anesthetic agent sevoflurane through dedicated devices for the sedation of ICU patients[12–15], with superior awakening and extubation times in comparison with current intravenous sedation agents (propofol and benzodiazepines). Notably, isoflurane and sevoflurane may possess important end-organ protective properties that are very relevant to the pathogenesis and resolution of ARDS. Several preclinical studies have shown that inhaled improves lung injury[16–19], reduces alveolar edema[19], and attenuates inflammation[20–22] in experimental ARDS. A first pilot randomized controlled trial from our team found that sevoflurane improved oxygenation, decreased inflammation and lung epithelial injury (as assessed by plasma soluble receptor for advanced glycation end-products (sRAGE)) in patients with ARDS[23], thus prompting the design and conduct of the "SEvoflurane for Sedation in ARds" (SESAR) randomized controlled trial (ClinicalTrials.gov Identifier: NCT04235608), funded through the 2018 French *Programme Hospitalier de Recherche Clinique National* and the 2019 European Society of Anaesthesiology (ESA) research project grant programs, which should start enrolling patients in April 2020. However, the conduct of a large multicenter randomized controlled trial is challenging and gaining evidence through SESAR may take too long to rapidly inform the potential benefits of using inhaled sedation in patients with ARDS due to COVID-19.

The current effects of inhaled sedation with isoflurane or sevoflurane in preclinical and clinical studies available to date[17,18,23–25] are summarized in the table below and compared to the general findings in patients with ARDS[26] and in patients with COVID-19-related ARDS[8].

		General findings in ARDS (Sweeney 2016)	Findings in Corona virus infection and ARDS (Huang 2020)	Effects of Inhaled Anaesthetics (isoflurane and/or sevoflurane) vs Intravenous Anaesthetics (controls)				
				Voigtsberger 2009 (rat model)	Ferrando 2013 (pig model)	Strosing 2016 (mouse model)	Kellner 2017 (rat model)	Jabaudon 2017 (human)
Pro-inflammatory cytokines	TNF-alpha	↑	↑	↓	↓	NA	NA	↓
	IL-1 beta	↑	↑	↓	↓	↓	NA	NS
	IL-6	↑	NA	↓	↓	NA	↓	↓
	IL-8	↑	↑	NA	↓	NA	NA	↓
	IL-10	↑	↑	NA	NA	NA	NS	NA
	MCP-I	↑	↑	↓	NA	NA	NS	NA
	CINC-I	↑	NA	↓	NA	NA	↓	NA
Other markers of lung injury	White blood cell count in alveoli	↑	NA	↓	↓	↓	↓	NA
	Lung fluid permeability/edema	↑	NA	↓	↓	NA	↓	NA
	Alveolar histological disruption	↑	NA	Maintained	NA	Maintained	NA	NA
	S-RAGE	↑	NA	NA	NA	NA	NA	↓
	Oxygenation	↓	↓	↑	↑	↑	↑	↑

NA: not assessed. NS: non-significant difference compared to the control intervention.

Research hypothesis and objectives

We hypothesized that inhaled sedation, either with isoflurane or sevoflurane, might be associated with improved clinical outcomes in patients with COVID-19-related ARDS, compared to intravenous sedation.

We therefore designed the "Inhaled Sedation for COVID-19-related ARDS" (ISCA) non-interventional, observational, multicenter study of data collected from the patients' medical records in order to:

- (1) assess the efficacy of inhaled sedation in improving a composite outcome of mortality and time off the ventilator at 28 days in patients with COVID-19-related ARDS, in comparison to a control group receiving intravenous sedation (**primary objective**),
- (2) investigate the effects of inhaled sedation, compared to intravenous sedation, on lung function as assessed by gas exchange and physiologic measures in patients with COVID-19-related ARDS (**secondary objective**),
- (3) report sedation practice patterns in critically ill patients during the COVID-19 pandemics (**secondary objective**).

This study will be performed in accordance with the Strengthening the Reporting of Observational studies in Epidemiology (STROBE) statement[27].

Study population

Description

Data will be collected from mechanically ventilated adult patients with suspected or confirmed COVID-19 and admitted to an ICU (or any other ICU-like setting that may be deployed as a result of the COVID-19 pandemics, such as in the operating room, post-anesthesia care unit, step-down unit or any COVID-19-specific unit set in response to the pandemics) of a participating center.

All data will be de-identified and collected from the patients' health records by study investigators or clinical research associates at each center, so that: 1) the risk for intensive care providers to be contaminated with the virus is minimized, as no data will be collected at patient bedside; 2) no additional work overload is needed from intensive care providers, as their staffing may be very challenged in this threatening pandemic situation.

Participating centers will be located in multiple countries such as France, Germany, Spain, Switzerland and the USA. The detailed list of participating centers is under construction but around 10 centers per country are expected. Each national investigator will be responsible for conforming to the regulatory and Ethics approval requirements in their specific country. Other countries might as well join this collaborative project along the way.

Patients will be followed up to 28 days after enrollment in the study, with day 0 being defined as the day of initiation of invasive mechanical ventilation.

Inclusion Criteria

- Adult patients (18 years old),
- Admitted to a participating ICU (or any other ICU-like setting that may be deployed as a result of the COVID-19 pandemics, such as in the operating room, post-anesthesia care unit, step-down unit or any COVID-19-specific unit set in response to the pandemics in a participating center),
- Requiring invasive mechanical ventilation,
- With suspected or confirmed COVID-19 on day 0.

Non-inclusion Criteria

None.

Recruitment procedure

Patients will be included retrospectively in the study by local investigators at each participating center.
As this is a non-interventional study, sedation practices will be those currently used as standard practices in participating centers, including both intravenous and inhaled sedation practices. The choice of the sedative agent (intravenous or inhaled), including type of and dosing of the agent, will be as per the treating clinicians at each center.

Study design and methods

Data collection

Data will be collected remotely from the (electronic) patient health record by investigators or clinical research associates at each center, so that:

1) the risk for intensive care providers to be contaminated with the virus is minimized, as no data will be collected at patient bedside

2) no additional work overload is needed from intensive care providers, as their staffing may be very challenged in this threatening situation.

In addition to data that are usually collected in the ICU patient's health record on a routine basis, relevant information on COVID-19, such as specific interventions that may be delivered in these patients (such as antiviral therapies, (hydroxy-) chloroquine or other specific therapies) will also be collected.
This strategy will allow the rigorous collection of most data relevant to major patient outcomes (survival, duration of mechanical ventilation and of ICU stay, indices of lung function, and patterns of sedation practices) while ensuring the safety of both intensive care providers and clinical research associates, and preventing any interaction to the priority care patients will receive in participating ICUs.
However, and as a result, some degree of missing data is anticipated for some variables that may be considered less relevant to collect from the (electronic) patient health record in the current pandemic context due to the surge in ICU patients with COVID-19.

Primary outcome measure

In this study, the primary outcome will be the number of days off the ventilator at 28 days (VFD28, for ventilator-free days to day 28), taking into account death as a competing event.
Ventilator-free days to day 28 are defined as the number of days from the time of initiating unassisted breathing to day 28 after intubation, assuming survival for at least two consecutive calendar days after initiating unassisted breathing and continued unassisted breathing to day 28. If a patient returns to assisted breathing and subsequently achieves unassisted breathing to day 28, VFDs will be counted from the end of the last period of assisted breathing to day 28. A period of assisted breathing lasting less than 24 hours and for the purpose of a surgical procedure will not count against the VFD calculation. If a patient was receiving assisted breathing at day 27 or died prior to day 28, VFDs will be zero. Patients transferred to another hospital or other health care facility will be followed to day 28 to assess this endpoint.

Secondary outcome measures

- All-cause mortality to day 28
- ICU-free days to day 28
- Duration of invasive mechanical ventilation to day 28; duration of controlled mechanical ventilation to day 28
- Physiological measures of lung function:
    - arterial hypoxemia, as assessed by the partial pressure of arterial oxygen-to-fraction of inspired oxygen ratio (PaO_2/FiO_2), on study days
    - partial pressure of arterial carbon dioxide ($PaCO_2$) on study days 1-7

    - inspiratory plateau pressure, compliance of the respiratory system, and driving pressure on study day 1-7
    - mode of mechanical ventilation (assisted versus controlled) used on study day 1-7
    - if available: 100 ms occlusion pressure (P0.1), a marker of respiratory drive, if available, on study day 1-7.
- Duration of vasopressor use from day 0 to day 28
- Need for, and duration of, renal replacement therapy from day 0 to day 28
- Need for, and duration of, any adjuvant intervention for severe ARDS (such as prone position, recruitment maneuvers, inhaled nitric oxide, inhaled epoprostenol sodium, high frequency ventilation, ECMO, and neuromuscular blockade) through day 7
- Need for, and duration of, continuous neuromuscular blockade for severe ARDS through day 28
- Type, duration, and modalities of sedation practices used in patients with COVID-19 during the pandemic

Origin and nature of the personal data collected

Data will be collected remotely from the (electronic) patient health record by investigators or clinical research associates at each center.

**Type of data**	**Data collected in the study (Yes/No)**
Identification with full name	No
Identification by study number	Yes<br>(per study design)
Other type of identification (please detail)	No
Health status (such as personal medical history, treatments etc.)	Yes<br>(all data already present in the patient's health record and that is relevant as part of his/her personal medical history will be collected)
Familial situation (such as married, single etc.)	No
Military situation	No
Education background (such as diploma, professional development etc.)	No

Professional situation (such as employment/unemployment, socio-professional status etc.)	No
Economic or financial situation (such as the level of income, social benefits etc.)	No
Consumption of goods and services (such as home assistance, transport etc.)	No
Lifestyle habits, behaviour (such as consumption of alcohol, of tobacco, sports etc.)	Yes<br>(consumption of alcohol and of tobacco, if known, is usually collected in the patient's health record as part of his/her personal medical history)
Social security/Health insurance number	No
Ethnic, religious, political, or any personal informations not listed above	No
identifying biological samples	No
Offences, convictions or security measures	No

Data flow and management

Data access

Data will be collected remotely from the patient's health record by investigators or clinical research associates at each center. The patient's health record will be either electronic or paper-based, depending on the current practice at each center.
De-identified data will be collected retrospectively, and either locally or remotely (depending on the current practices at each center in response to self-isolation and social distancing measures due to the pandemics). Data will be collected by trained research coordinators and/or investigators from each participating center, using REDCap electronic data capture tools hosted at CHU Clermont-Ferrand, France[28]. REDCap (Research Electronic Data Capture) is a secure, web-based application designed to support data capture for research studies, providing: (1) an intuitive interface for validated data entry; (2) audit trails for tracking data manipulation and export procedures; (3) automated export procedures for seamless data downloads to common statistical packages; and (4) procedures for importing data from external sources.
De-identified data will be accessible to the coordinating and national investigators, as well as to the study's methodologist (BP).

Data source

Any original document or object that proves the existence or accuracy of a data or fact recorded in the course of the research is defined as a source document.

Data privacy

In accordance with the legislative provisions in force (Articles L.1121-3 and R.5121-13 of the French Public Health Code), persons with direct access to source data shall take all necessary precautions to ensure the confidentiality of information related to investigational medicinal products, research, persons involved in the research, and in particular with regard to their identity and the results obtained. These persons, like the investigators themselves, shall be subject to professional confidentiality and secrecy. During or at the end of the research, the data collected on suitable persons and transmitted to the sponsor by the investigators shall be rendered anonymous. Under no circumstances should the names or addresses of the persons concerned be made clear.
Each patient included in the study will receive a registration code consisting of 3 numbers, corresponding to the patient's inclusion number in the study as generated by REDCap.

Provisional study timeline

- Submission to the Ethics committee:
    - France (Comité d'Ethique de la SFAR-CERAR): March 2020; Submission CNIL / CCTIRS: April 2020
    - Germany: April 2020
    - Spain: April 2020
    - Switzerland: April 2020
    - USA: April 2020
- Approval by Ethics committee:
    - France: IRB 00010254-2020-050 (Comité d'éthique pour la Recherche en Anesthésie-Réanimation - CERAR, SFAR) (April 6, 2020); Approval by CNIL / CCTIRS: April 2020
    - Germany: April 27, 2020 (German Ethikkommission der medizinischen Fakultät der Christian-Albrechts-Universität zu Kiel; Approval D 471/20)
    - Spain: April 30, 2020 (COMITE ETICO DE INVESTIGACION CON MEDICAMENTOS; SAS/3470/2009, CHU-ISO-2020-01 (ISCA))
    - Switzerland: June 11 2020 (*add details*)
    - USA: May 2020 (*add details*)
- Study recruitment starts: May 11, 2020
- Duration of recruitment: 6 months
- Duration of participation for each patient: 28 days
- Planned study completion: November 2020
- End of study report: December 2020

- Total duration of the research: 9 months

Summary table of patient follow-up

	Enrollment	Day 0	Day 1	Day 2	Day 3	Day 4	Day 5	Day 6	Day 7	Day 28
Participating Center										
Inclusion Criteria										
Demographics & Medical History										
Day -1										
ICU admission & initiation of mechanical ventilation										
Daily Data_Days 0-7										
Follow-Up Day 28										

Link to the eCRF (draft version - the final eCRF will be developed using REDCap):
https://docs.google.com/spreadsheets/d/16kiwHTXd_kbuToE1ZIpNWIOsdgbu5Gqn222_DPCl-Ic/edit?usp=sharing

Statistics and data analysis

Sample size estimation

In this study, the event of interest (primary outcome) is the number of days off the ventilator at 28 days (VFD28, for ventilator-free days to day 28) and the competing event is death.
Given the limited data available to date on clinical outcomes of patients with COVID-19-related ARDS[1–4,7,29], and given that estimates for the effects of inhaled sedation on major outcomes are scarce and only available in patients with non-COVID-19-related ARDS[23], it seems difficult to give a robust sample size estimation from an expected difference in the primary outcome (VFD28) between patients with COVID-19-related ARDS receiving inhaled sedation (with either isoflurane or sevoflurane) and those receiving intravenous sedation (whatever the sedative agent).
However, it is anticipated that if approximately 5-10 centers from each of the participating countries (France, Germany, Spain, Switzerland, USA) include 10-15 patients each, a total number of 150-450 patients could be rapidly enrolled. Although not supported by precise power calculation, this potential sample size is considered very relevant to evaluate the primary endpoint of the study, while exploring all secondary endpoints.

Data analysis

General aspects

All analyses will be performed with Stata software (version 15, StataCorp, College Station, USA) or R version 3.6.3 (R Foundation for Statistical Computing, Vienna, Austria) by Bruno Pereira, PhD Biostatistics, Biostatistics unit, CHU Clermont-Ferrand, France. The primary analysis will be performed prior to analyses of secondary outcome measures, and followed by subgroup analyses on the primary outcome and the most important secondary outcomes.

Continuous variables will be presented as means and standard-deviations (as medians and quartiles, otherwise) and will be compared with the use of the unpaired t test or the Mann-Whitney U test when appropriate. The Shapiro-Wilk test will be used to assess normality, and the Fisher-Snedecor test to assess homoscedasticity. Categorical data will be presented as exact number and percentage and will be compared using unadjusted Chi-squared or Fisher's exact tests.

A two-sided P value of less than 0.05 will be considered for statistical significance of all analyses. As described by some authors[30], systematic correction of type I error will not be applied. As presented by Feise in 2002[31], "it must be careful to focus not only upon statistical significance (adjusted or not), but also upon the quality of the research within the study and the magnitude of difference".

Primary analysis

The primary outcome is days off the ventilator at 28 days (VFD28, for ventilator-free days to day 28), taking into account death as a competing event.

Therefore, the primary analysis will be based on a mixture of generalized gamma distributions to concatenate the overall frequency and distribution of the times.

Exploratory sequential analyses will be performed each time 100 patients are enrolled. Particular attention will be given on measures of safety and efficacy. The results will be expressed as absolute differences (with 95% confidence intervals) for adverse events and as standardized mean (or median) differences (with 95% confidence intervals) for the primary endpoint.

Secondary analysis

The analysis of the primary outcome will be completed by multivariable analysis using a generalized gamma distribution mixed model to take into account: (1) fixed effects covariates determined according to univariate results and clinical relevance, and (2) center as random-effects (to measure between- and within-center variability). Results will be expressed as regression coefficients and 95% confidence intervals (CI). According to usual recommendations, the interactions between possible predictive factors will also be tested before considering unplanned subgroup analyses.

Because in observational non-randomized studies the choice of treatment is generally influenced by the characteristics of the patient, the subjects treated with

inhaled sedation, either with isoflurane or sevoflurane, and those treated with intravenous sedation could be different. Therefore, to adjust for these differences and to be as close as possible to the gold standard characteristics of a randomized controlled trial, a propensity score (PS) can be carried out. The PS corresponds to the probability for a patient to receive the treatment according to its characteristics and different methods, such as inverse probability of treatment weighting (IPTW), can be used to estimate this probability. Each participant is assigned an inverse weighting of the probability of receiving inhaled or intravenous sedation, estimated by the PS. Thus, the weight of patients who are highly likely to receive inhaled sedation based on their observable characteristics will be reduced, and that of patients who are unlikely to receive inhaled sedation will be increased. By doing so, inhaled and intravenous sedation groups will be made more comparable. The validity of the matching will be tested by analysis of standardized differences (|d|), with |d|> 0.2 considered an imbalance. As in multivariable models, variables that will be integrated into the propensity score will include (1) fixed effects covariates determined according to univariate results and clinical relevance (such as age, severity of baseline illness as evaluated by various scores (eg, SOFA, APACHE 2 or 3) that will be concatenated into a z-score as needed, the degree of ARDS severity (PaO_2/FiO_2 <100 mmHg), and the presence of shock at study entry), and (2) center as random-effects (to measure between- and within-center variability).

A particular focus will be paid on descriptive analysis of sedation practice patterns.

As for the analysis of primary endpoint, matching using IPTW may be considered for the following secondary analyses.

Censored data will be estimated using Kaplan-Meier approach and will be compared using log-rank test in univariate analysis and marginal Cox proportional hazard regression in multivariable analysis, (1) to take into account adjustment on possible confounding covariates selected according to univariable results and clinical relevance, and (2) to consider within- and between-center variability. Results will be expressed as hazard-ratios with 95% CI and proportional-hazard assumption will be verified using the Schoenfeld test and plotting residuals.

Continuous parameters outcomes will be compared between groups using Student's t-test or Mann-Whitney U test. Normality will be studied by the Shapiro-Wilk test and homoscedasticity using the Fisher-Snedecor test. Multivariable analyses will be performed with linear mixed models to take into account: (1) fixed effects covariates determined according to univariate results and to clinical relevance and (2) center as random-effects. When appropriate, logarithmic transformation will be considered to achieve normality of dependent outcome. Results will be expressed as regression coefficients and 95% CI.

Categorical variables will be analysed using chi-squared test or Fisher's exact test, as appropriate. For multivariable analysis, adjusted analyses will be performed with the use of random-effects robust Poisson generalized linear model to take into account within- and between-center variability with center as random-effect. Results will be expressed as Relative Risks and 95% CI.

Longitudinal analysis using mixed models will be performed in order to study fixed effects group, time-point evaluation, and their interaction taking into account between- and within-subject variability.

Method for missing data

To put significant results into perspective, a sensitivity analysis will be conducted to measure the impact of missing data. When appropriate, additional analyses will then be performed according to the statistical nature of missing data (missing at random or not).

Missing primary outcome data

We do not expect missing data for the primary outcome measure and only complete case-analysis will be performed. Furthermore, death will be considered as a competing risk.

Missing secondary outcome data

There will be no imputation of missing data.

Subgroups

Subgroup analyses will be performed based on:

1. Patients without ARDS on day 0, as defined by the Berlin definition criteria[32]
2. Patients with mild ARDS (PaO_2/FiO_2 <300 mmHg) on day 0
3. Patients with severe ARDS (PaO_2/FiO_2 <100 mmHg) on day 0
4. Patients receiving a specific agent for inhaled sedation: isoflurane or sevoflurane
5. Patients receiving a specific agent for intravenous sedation: propofol, midazolam, dexmedetomidine or other(s)
6. Patients with shock (defined as the need for intravenous vasopressor infusion to maintain arterial pressure) on day 0
7. Patients with “longer” duration of inhaled sedation, as defined as the need for inhaled sedation for ≥5 days
8. Patients included in an “ICU-like” setting (any other hospital setting that may be deployed to manage critically ill patients as a result of the COVID-19 pandemics, such as in the operating room, post-anesthesia care unit, step-down unit or any COVID-19-specific unit set in response to the pandemics)

Study costs and funding

The costs of the ISCA study will be those related to data collection by clinical research associates at each center.

It is estimated that an average of 4.5 hours per patient would be needed for a clinical research associate or a research nurse to fill the eCRF from data already present in the patient’s health record.

Given a total of 400 patients to be enrolled in the ISCA study, the maximal total cost of the study is **61,200€** (cost for 4.5 hours of clinical research associate/research nurse staff per patient x 400 patients x 34€/hour).

This funding will be centralized and managed among participating centers by the study sponsor (CHU Clermont-Ferrand), in a way that the costs related to the study

will be paid in proportion to the inclusions made by each center, for example each time 10 patients are enrolled.
A funding of **40,000€** has already been obtained through the Sedana Foundation Research Grant 2020. The funder(s) will have no influence on the study design, conduct, analysis, and report.

Risk management strategy

During the whole study period, the coordinating investigator and the National investigators will meet twice a month to discuss current progress in patient enrollment and will be able to "activate" this risk management strategy whenever they deem it necessary.

Rules for publication and authorship

All study sites including patients will be acknowledged, and all investigators at these sites will appear with their names under the "ISCA investigators" in an Appendix to the final manuscript.

Authorship for publication will be granted according to the Vancouver definitions to a writing committee composed of the coordinating investigator and the National investigators. All publications will include the mention "for the ISCA study group" after the last author.

List of participating centers *- (Under construction) -*

Name	City	Country	Name and contact of the local principal investigator
Department of Anesthesiology and Critical Care, Pitié-Salpêtrière Hospital, AP-HP	Paris	France	Pr Jean-Michel CONSTANTIN jean-michel.constantin@aphp.fr
Department of Perioperative Medicine, CHU Clermont-Ferrand	Clermont-Ferrand	France	Dr Raiko BLONDONNET rblondonnet@chu-clermont-ferrand.fr
CH Privé de la Loire	Saint-Etienne	France	Dr Pierre BOUCHER pierreboucher85@yahoo.fr
CH Dunkerque	Dunkerque	France	Dr Caroline VARILLON caroline.varillon@ch-dunkerque.fr
Other centers to be determined			

Department of Anesthesiology and Intensive Care Medicine, University Medical Center Schleswig-Holstein, Campus Kiel	Kiel	Germany	Dr Tobias BECHER<br>tobias.becher@uksh.de
Universitätsklinik Oldenburg	Oldenburg	Germany	Dr Anja DIERS<br>diers.anja@klinikum-oldenburg.de<br>Dr Ulf GÜNTER<br>guenther.ulf@klinikum-oldenburg.de
Universitätsklinikum Würzburg	Würzburg	Germany	Dr Christoph LOTZ<br>lotz_c@ukw.de
Universitätsklinikum Bochum	Bochum	Germany	Dr Martin Bellgardt<br>martin.bellgardt@gmx.de
Universitätsklinikum Greifswald	Universitätsklinikum Greifswald	Germany	Dr Sven-Olaf KUHN<br>sven-olaf.kuhn@med.uni-greifswald.de
Universitätsklinikum Schleswig-Holstein, Campus Lübeck	Lübeck	Germany	Dr Patrick KELLNER<br>patrick.kellner@uksh.de
Klinikum Leverkusen	Leverkusen	Germany	Dr Christian MEY<br>christian.mey@klinikum-lev.de
Krankenhaus Korbach	Korbach	Germany	Dr Michael TÜBBEN<br>m.tuebben@krankenhaus-korbach.de
Krankenhaus Vechta	Vechta	Germany	D. Christian HÖHNNEMANN<br>christian.hoenemann@kh-vec.de
Universitätsklinikum Saarland	Hombourg	Germany	Dr Andreas MEISER<br>andreas.meiser@uks.eu
Marienhospital Osnabrück	Osnabrück	Germany	Dr Thorsten HEUTER<br>thorsten.heuter@niels-stensen-kliniken.de
Klinikum Saarbrücken	Saarbrücken	Germany	Dr Konrad SCHWARZKOPF<br>kschwarzkopf@klinikum-saarbruecken.de
Other centers to be determined			
Department of Anesthesiology and Intensive Care, Hospital Clínico Universitario de Valencia	Valencia	Spain	Dr Rafael BADENES<br>rafaelbadenes@gmail.com
Other centers to be determined			

Institute of Anaesthesiology, University Hospital Zurich	Zurich	Switzerland	Pr Beatrice BECK-SCHIMMER<br>beatrice.beckschimmer@uzh.ch<br>Dr Martin SCHLAPFER<br>martin.schlaepfer@usz.ch
Other centers to be determined			
Department of Anesthesia, Critical Care & Pain Medicine, Beth Israel Deaconess Medical Center, Boston	Boston	USA	Dr Brian P. O'GARA<br>bpogara@bidmc.harvard.edu

References

1. Murthy, S., Gomersall, C. D. & Fowler, R. A. Care for Critically Ill Patients With COVID-19. *JAMA* (2020) doi:10.1001/jama.2020.3633.
2. Liao, X., Wang, B. & Kang, Y. Novel coronavirus infection during the 2019-2020 epidemic: preparing intensive care units-the experience in Sichuan Province, China. *Intensive Care Med.* **46**, 357–360 (2020).
3. Grasselli, G., Pesenti, A. & Cecconi, M. Critical Care Utilization for the COVID-19 Outbreak in Lombardy, Italy: Early Experience and Forecast During an Emergency Response. *JAMA* (2020) doi:10.1001/jama.2020.4031.
4. Vincent, J.-L. & Slutsky, A. S. Coronavirus: just imagine…. *Crit. Care* **24**, 90 (2020).
5. Matthay, M. A., Aldrich, J. M. & Gotts, J. E. Treatment for severe acute respiratory distress syndrome from COVID-19. *The Lancet Respiratory Medicine* (2020) doi:10.1016/S2213-2600(20)30127-2.
6. Ramanathan, K. *et al.* Planning and provision of ECMO services for severe ARDS during the COVID-19 pandemic and other outbreaks of emerging infectious diseases. *The Lancet Respiratory Medicine* (2020) doi:10.1016/S2213-2600(20)30121-1.
7. Arentz, M. *et al.* Characteristics and Outcomes of 21 Critically Ill Patients With COVID-19 in Washington State. *JAMA* (2020) doi:10.1001/jama.2020.4326.
8. Xu, Z. *et al.* Pathological findings of COVID-19 associated with acute respiratory distress syndrome. *Lancet Respir Med* (2020) doi:10.1016/S2213-2600(20)30076-X.
9. Cao, B. *et al.* A Trial of Lopinavir-Ritonavir in Adults Hospitalized with Severe Covid-19. *N. Engl. J. Med.* (2020) doi:10.1056/NEJMoa2001282.
10. Wu, C. *et al.* Risk Factors Associated With Acute Respiratory Distress Syndrome and Death in Patients With Coronavirus Disease 2019 Pneumonia in Wuhan, China. *JAMA Intern. Med.* (2020) doi:10.1001/jamainternmed.2020.0994.
11. Yang, X. *et al.* Clinical course and outcomes of critically ill patients with SARS-CoV-2 pneumonia in Wuhan, China: a single-centered, retrospective, observational study. *Lancet Respir Med* (2020) doi:10.1016/S2213-2600(20)30079-5.
12. Bisbal, M. *et al.* [Efficacy, safety and cost of sedation with sevoflurane in intensive care unit]. *Ann. Fr. Anesth. Reanim.* **30**, 335–341 (2011).
13. Mesnil, M. *et al.* Long-term sedation in intensive care unit: a randomized comparison between inhaled sevoflurane and intravenous propofol or midazolam. *Intensive Care Med.* **37**, 933–941 (2011).
14. Jerath, A., Parotto, M., Wasowicz, M. & Ferguson, N. D. Volatile Anesthetics. Is a New Player Emerging in Critical Care Sedation? *Am. J. Respir. Crit. Care Med.* **193**, 1202–1212 (2016).
15. Perbet, S. *et al.* A pharmacokinetic study of 48-hour sevoflurane inhalation using a disposable delivery system (AnaConDa®) in ICU patients. *Minerva Anestesiol.* **80**, 655–665 (2014).
16. Reutershan, J., Chang, D., Hayes, J. K. & Ley, K. Protective effects of isoflurane pretreatment in endotoxin-induced lung injury. *Anesthesiology* **104**, 511–517 (2006).
17. Voigtsberger, S. *et al.* Sevoflurane ameliorates gas exchange and attenuates

lung damage in experimental lipopolysaccharide-induced lung injury. *Anesthesiology* **111**, 1238–1248 (2009).
18. Ferrando, C. *et al.* Sevoflurane, but not propofol, reduces the lung inflammatory response and improves oxygenation in an acute respiratory distress syndrome model: A randomised laboratory study. *Eur. J. Anaesthesiol.* **30**, 455–463 (2013).
19. Schläpfer, M. *et al.* Sevoflurane reduces severity of acute lung injury possibly by impairing formation of alveolar oedema. *Clin. Exp. Immunol.* **168**, 125–134 (2012).
20. Suter, D. *et al.* The immunomodulatory effect of sevoflurane in endotoxin-injured alveolar epithelial cells. *Anesth. Analg.* **104**, 638–645 (2007).
21. Steurer, M. *et al.* The volatile anaesthetic sevoflurane attenuates lipopolysaccharide-induced injury in alveolar macrophages. *Clin. Exp. Immunol.* **155**, 224–230 (2009).
22. Du, X. *et al.* Isoflurane promotes phagocytosis of apoptotic neutrophils through AMPK-mediated ADAM17/Mer signaling. *PLoS One* **12**, e0180213 (2017).
23. Jabaudon, M. *et al.* Sevoflurane for Sedation in Acute Respiratory Distress Syndrome. A Randomized Controlled Pilot Study. *Am. J. Respir. Crit. Care Med.* **195**, 792–800 (2017).
24. Strosing, K. M. *et al.* Inhaled Anesthetics Exert Different Protective Properties in a Mouse Model of Ventilator-Induced Lung Injury. *Anesth. Analg.* **123**, 143–151 (2016).
25. Kellner, P. *et al.* Sevoflurane Abolishes Oxygenation Impairment in a Long-Term Rat Model of Acute Lung Injury. *Anesth. Analg.* **124**, 194–203 (2017).
26. Sweeney, R. M. & McAuley, D. F. Acute respiratory distress syndrome. *Lancet* **388**, 2416–2430 (2016).
27. von Elm, E. *et al.* The Strengthening the Reporting of Observational Studies in Epidemiology (STROBE) statement: guidelines for reporting observational studies. *J. Clin. Epidemiol.* **61**, 344–349 (2008).
28. Harris, P. A. *et al.* Research electronic data capture (REDCap)--a metadata-driven methodology and workflow process for providing translational research informatics support. *J. Biomed. Inform.* **42**, 377–381 (2009).
29. Onder, G., Rezza, G. & Brusaferro, S. Case-Fatality Rate and Characteristics of Patients Dying in Relation to COVID-19 in Italy. *JAMA* (2020) doi:10.1001/jama.2020.4683.
30. Rothman, K. J. & Greenland, S. Modern epidemiology. 2nd. *Philadelphia, PA, Lippencott-Raven Publishers* (1998).
31. Feise, R. J. Do multiple outcome measures require p-value adjustment? *BMC Med. Res. Methodol.* **2**, 8 (2002).
32. ARDS Definition Task Force *et al.* Acute respiratory distress syndrome: the Berlin Definition. *JAMA* **307**, 2526–2533 (2012).
