# Supplementary material for: Inhaled Sedation in Patients with COVID-19-Related Acute Respiratory Distress Syndrome: An International Retrospective Study
Source: J Clin Med. 2022 Dec 20;12(1):12. doi: 10.3390/jcm12010012 (PMC9821008; doi:10.3390/jcm12010012)
Supplement: Supplementary file 1 [file jcm-12-00012-s001.zip › Supplementary Materials.pdf]

Supplementary Materials

Complete list of the ISCA Study Group Members

Dominique Morand, Elodie Caumon, Lucile Borao, Nathalie Bourguignon, Raiko Blondonnet, Bruno Pereira, Randy Coupet, Matthieu Jabaudon (Clermont-Ferrand, France); Martin Schläpfer, Sabine Kern, Beatrice Beck-Schimmer (Zurich, Switzerland); Thomas A. Neff, Nadine Hungerbuehler (Muensterlingen, Switzerland); Pierre Boucher, Taïssia Lelekov-Boissard (Saint-Etienne, France); Pierre Bailly, Erwan L'Her (Brest, France); Martin Bellgardt (Bochum, Germany); Rafael Badenes, Jose Carbonell, Berta Monleón (Valencia, Spain); Tobias Becher, Nina Schulz-Ruhtenberg, Corinna Buchholz, Risto Radczewski (Kiel, Germany); Caroline Varillon (Dunkerque, France); Jean-Michel Constantin (Paris, France); Ronny Munoz Acun, Thy Nguyen, Brian O'Gara (Boston, USA).

Supplementary Figure Legends:

- **Figure S1.** Modes of invasive mechanical ventilation on study days 0–7 in the patients who received inhaled sedation for at least 24 h (inhaled sedation group) and those who received intravenous sedation only (intravenous sedation group) through day 28.
- **Figure S2.** Adjunctive therapies for acute respiratory distress syndrome used on study days 0–7 in the patients who received inhaled sedation for at least 24 h (inhaled sedation group) and those who received intravenous sedation only (intravenous sedation group) through day 28. RM: recruitment maneuvers; NMB: neuromuscular blockade; NO: inhaled nitric oxide; ECMO: extracorporeal membrane oxygenation.
- **Figure S3.** Agents of sedation used on study days 0–7 in the patients who received inhaled sedation for at least 24 h (inhaled sedation group) and those who received intravenous sedation only (intravenous sedation group) through day 28.
- **Figure S4.** Components of the ABCDEF bundle implemented on study days 0–7 in the patients who received inhaled sedation for at least 24 h (inhaled sedation group) and those who received intravenous sedation only (intravenous sedation group) through day 28. The "C" component of the bundle (choice of analgesia and sedation) is not shown as it was not captured as a binary variable; details on analgesia and sedation are provided elsewhere.

Supplementary Tables

- **Table S1.** List of participating centers, number of patients included per center, and number of patients who died by day 28 in each center.
- **Table S2.** Demographics and medical history.
- **Table S3.** Univariate analysis of the primary endpoint using demographic variables, variables from past medical history, variables from the day before inclusion, and variables from the day of inclusion.
- **Table S4.** Baseline variables integrated in the propensity score that was used to predict probability of receiving inhaled sedation or not, and derived from the fitted regression model (logistic regression).
- **Table S5.** Selected characteristics (demographics, medical history, data from study day 0) according to treatment assignment (inhaled versus intravenous sedation) in propensity-matched patients.
- **Table S6.** Primary and secondary endpoints according to treatment assignment (inhaled versus intravenous sedation) in propensity-matched patients.
- **Table S7.** Sensitivity analysis of the primary endpoint (ventilator-free days through day 28) when defining groups based on the length of inhaled sedation received (≥2, ≥3, ≥4, ≥5, ≥7 or ≥10 days).
- **Table S8.** Subgroup unadjusted analysis of the primary endpoint (ventilator-free days through day 28).
- **Table S9.** Mechanical ventilation data and adjunctive ARDS therapies received during the first seven days after inclusion.
- **Table S10.** Physiological measures of lung function and ventilator settings through day 7 in patients receiving inhaled sedation and those receiving intravenous sedation only.
- **Table S11.** Sedation practices and measures from the ABCDEF bundle implemented during the first seven days after inclusion.

Materials and Methods

Study Design, Population, and Data Collection

This retrospective, observational, multicenter study was conducted at 10 university and non-university hospitals in France, Germany, Spain, Switzerland, and the United States **(Table S1, Supplementary Materials)**. This study was registered on ClinicalTrials.gov (identifier, NCT04383730) on May 12, 2020, prior to patient enrollment and performed in accordance with the STrengthening the Reporting of OBservational studies in Epidemiology (STROBE) statement (**Supplementary Digital Content 1**) [1]. The study protocol is provided in the **Supplementary Digital Content 2**.

Consecutive adult patients admitted to a participating ICU (or any other ICU-like setting deployed in response to the COVID-19 pandemic, such as an operating room, post-anesthesia care unit, step-down unit or any COVID-19-specific unit) for severe COVID-19 (suspected or confirmed) and requiring invasive mechanical ventilation and sedation between March 2020 and May 2021 were included. There were no exclusion criteria. As this was a non-interventional study, sedation practices were those routinely used in the participating centers; the choice and doses of the sedative agent (intravenous or inhaled) were as per each center's clinical standards. The patients were divided into two groups: those who received inhaled sedation for at least 24 hours and those who received only intravenous sedation within 28 days of enrollment.

De-identified, clinico-biological data routinely recorded in the patient health record, and relevant information on COVID-19, including specific therapies, were retrospectively collected by investigators or clinical research associates in REDCap electronic data capture tools (Vanderbilt University) hosted at CHU Clermont-Ferrand, France [2]. The patients were followed up to 28 days after enrollment in the study, with day 0 (baseline) defined as the day when patients were admitted to a participating center, invasively ventilated, and receiving sedation.

Ethics and Consent

The study protocol was approved by ethics committees from France (Comité d'Ethique pour la Recherche en Anesthésie et Réanimation, Société Française d'Anesthésie et de Réanimation, IRB 00010254-2020-050), Germany (Ethikkommission der medizinischen Fakultät der Christian-Albrechts-Universität zu Kiel, approval D471/20), Spain (Comité de Ética de la Investigación con Medicamentos del Hospital Clínico Universitario de Valencia, approval SAS/3470/2009), Switzerland (Business Administration System for Ethics Committees No. 2020-01448), and the United States (Beth Israel Deaconess Medical Center, 2020P000326). Signed informed consent for this retrospective study was waived by ethics committees. All data were collected anonymously.

Endpoints

Primary Endpoint

The primary outcome was the number of ventilator-free days through day 28, considering death as a competing event (the patients who died within 28 days were assigned zero ventilator-free days). A period of assisted breathing lasting less than 24 hours or for surgical purposes was not considered for ventilator-free days calculation.

Secondary Endpoints

The secondary outcome measures were: all-cause mortality at day 28; the number of ICU-free days through day 28; the duration of invasive mechanical ventilation through day 28; the duration of controlled mechanical ventilation through day 28; physiological measures of lung function and ventilator settings on study days 0–7; the duration of vasopressor support and continuous neuromuscular blockade and the need for and duration of renal replacement therapy through day 28; the use of prone position and adjuvant interventions for severe ARDS (e.g., recruitment maneuvers, inhaled nitric oxide, high frequency ventilation, and extracorporeal membrane oxygenation) through day 7; and the

type, duration, and modalities of sedation practices on study days 0–7 (including the implementation of measures from the ABCDEF bundle) [3] and through day 28.

Statistical analyses

Analyses were performed with Stata version 15 (StataCorp) and *R: a language and environment for statistical computing* (v 3.6.3) [4]. Statistical significance was established by a P value of 0.05 using two-sided hypothesis tests. We did not correct for multiple comparisons and results should be interpreted as exploratory. No missing data were imputed.

For the primary analysis of ventilator-free days through day 28, zero-inflated negative binomial regression was performed, and the results were expressed with 95% confidence intervals (CI) as the odds ratio (OR) for having zero ventilator-free days through day 28 and the incidence rate ratio (IRR) for the number of ventilator-free days through day 28 when not zero. We also analyzed the primary endpoint using zero-inflated negative binomial regression after adjustment for the site (as a random effect) and the covariates determined according to the univariate results and clinical relevance (age, a medical history of arterial hypertension, and the partial pressure of arterial oxygen [PaO_2] to fraction of inspired oxygen [FiO_2] ratio, the need for vasopressor support, the documentation of an agitation-sedation scale by the bedside nurse, and serum creatinine at baseline). To maximize the number of complete cases in the multivariable analysis, these covariates were not included when the number of available individuals was <75% of the total study population. Subgroup analyses of the primary endpoint were performed after the interaction between the treatment (inhaled vs. intravenous sedation) and the predefined subgroups was tested as follows: in the patients with mild ARDS (PaO_2/FiO_2 <300 mmHg) at baseline; in the patients with severe ARDS (PaO_2/FiO_2 <100 mmHg) at baseline; in the patients receiving a specific agent for inhaled sedation (isoflurane or sevoflurane) at baseline; in the patients receiving a specific agent for intravenous sedation (propofol, midazolam, dexmedetomidine or other) at baseline; in the patients with shock (defined as the need for intravenous vasopressor infusion to maintain arterial pressure) at baseline; in the patients who required inhaled sedation for 5 days or more; and in the patients included in an ICU-like setting (i.e., any non-ICU setting that may be deployed to manage critically ill patients as a result of the COVID-19 pandemic, such as an operating room, a post-anesthesia care unit, or a step-down unit). To account for selection bias and potential confounders in the evaluation of the primary endpoint, propensity score matching was performed using the predicted probability of the group (inhaled or intravenous sedation) derived from the fitted logistic regression model regression, with the covariates identified as clinically relevant after the univariate analysis included in the propensity score model. The propensity score was used to compare the variables and outcomes in a cohort of propensity-matched patients and as a covariate in the multivariable analyses. Because we initially constructed the inhaled sedation and intravenous sedation groups based on whether they had received at least 24 hours of inhaled sedation or only received intravenous sedation through day 28, sensitivity analyses of the primary endpoint were also performed considering distinct durations of inhaled sedation (≥2, ≥3, ≥4, ≥5, ≥7, or ≥10 days) for the patients in the inhaled sedation group.

The continuous parameters were compared between the groups using Student's t-test or the Mann-Whitney U test after normality was assessed by the Shapiro-Wilk test and homoscedasticity using the Fisher-Snedecor test. The results were expressed as regression coefficients and 95% CI. When appropriate, logarithmic transformation was used to achieve normality of the dependent outcome. Longitudinal analysis of repeated data (such as the measures of lung function through day 7) was performed using mixed models to study the fixed effects group, the time point evaluation, and their interaction considering between- and within-subject variability. The categorical variables were analyzed using the chi-squared test or Fisher's exact test, as appropriate, and the results were expressed as relative risks and 95% CI.

Given that estimates for the effects of inhaled sedation on major outcomes are scarce and available only in a few patients with non-COVID-19 ARDS [5], it was impossible to

estimate a robust sample size from an expected difference in the primary outcome between the patients receiving inhaled sedation and those receiving intravenous sedation. However, it seemed that, given the number of centers involved, enrolling 150–450 patients would be feasible and relevant to assess the primary endpoint while exploring the secondary endpoints.

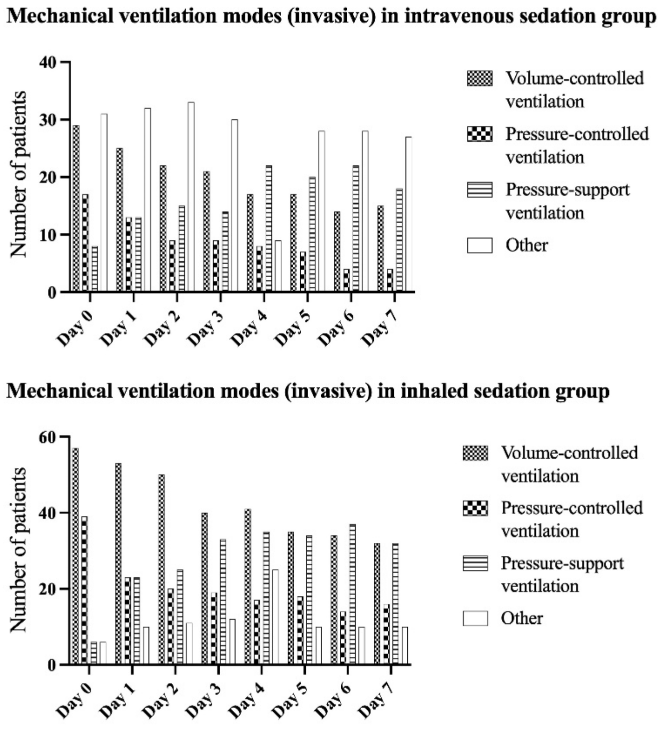

Figure S1. Modes of invasive mechanical ventilation on study days 0–7 in the patients who received inhaled sedation for at least 24 h (inhaled sedation group) and those who received intravenous sedation only (intravenous sedation group) through day 28.

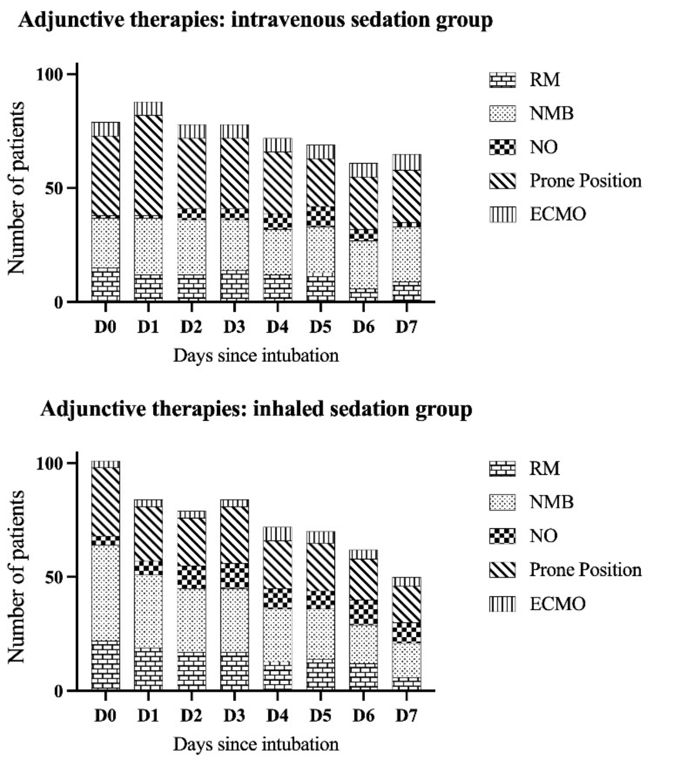

Figure S2. Adjunctive therapies for acute respiratory distress syndrome used on study days 0–7 in the patients who received inhaled sedation for at least 24 h (inhaled sedation group) and those who received intravenous sedation only (intravenous sedation group) through day 28. RM: recruitment maneuvers; NMB: neuromuscular blockade; NO: inhaled nitric oxide; ECMO: extracorporeal membrane oxygenation.

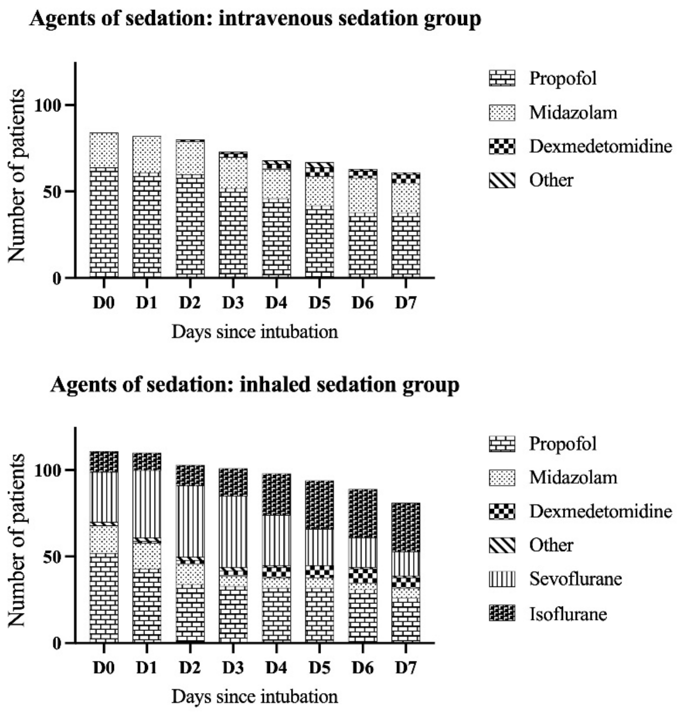

Figure S3. Agents of sedation used on study days 0–7 in the patients who received inhaled sedation for at least 24 h (inhaled sedation group) and those who received intravenous sedation only (intravenous sedation group) through day 28.

ABCDEF bundle: intravenous sedation group

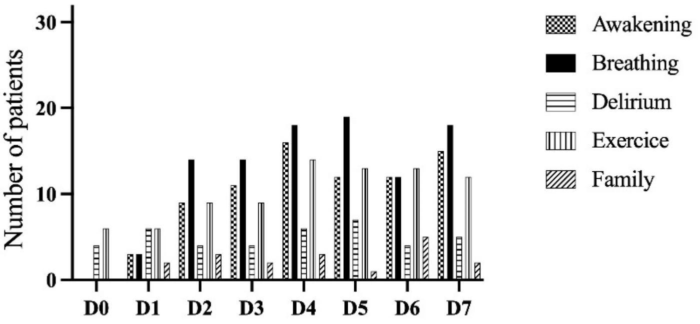

ABCDEF bundle: inhaled sedation group

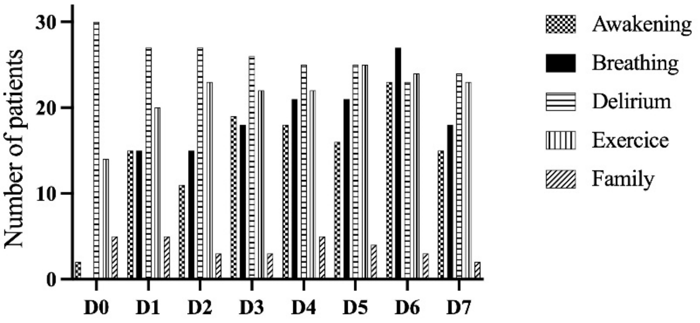

Figure S4. Components of the ABCDEF bundle implemented on study days 0–7 in the patients who received inhaled sedation for at least 24 h (inhaled sedation group) and those who received intravenous sedation only (intravenous sedation group) through day 28. The "C" component of the bundle (choice of analgesia and sedation) is not shown as it was not captured as a binary variable; details on analgesia and sedation are provided elsewhere.

Table S1. List of participating centers, number of patients included per center, and number of patients who died by day 28 in each center.

Center	Principal Investigator	Patients included, n	Patients who died by day 28, n (%)
University Clinic Hospital, Valencia, Spain	Rafael Badenes	12	2 (17)
Brest Regional University Hospital, France	Pierre Bailly	19	3 (16)
Clermont-Ferrand University Hospital, France	Matthieu Jabaudon	17	12 (71)
Dunkerque Hospital, Dunkerque, France	Caroline Varillon	7	4 (57)
Ramsay Santé Private Hospital de la Loire, Saint-Etienne, France	Pierre Boucher	24	7 (29)
University Hospital of Ruhr, Bochum, Germany	Martin Bellgardt	8	4 (50)
University Medical Center Schleswig-Holstein, Campus Kiel, Kiel, Germany	Tobias Becher	10	6 (60)
University Hospital, Zurich, Switzerland	Martin Schläpfer	52	11 (21)
Cantonal Hospital Münsterlingen, Münsterlingen, Switzerland	Thomas A. Neff	40	16 (40)
Beth Israel Deaconess Medical Center, Boston, USA	Brian O'Gara	7	6 (86)

Percentages may not sum to 100% due to rounding.

Table S2. Demographics and medical history.

Variable	No. of Available Individuals	Whole Cohort (n=196)	Inhaled Sedation (n=111)	Intravenous Sedation (n=85)	*P* Value*
Demographics					
Age, mean ± SD	196	66 ± 9	67 ± 10	66 ± 9	0.37
Men, n (%)	194	150 (77)	84 (76)	66 (79)	0.71
Body mass index, median (IQR), kg/m²	193	28.1 [24.8; 31.8]	28.7 [24.8; 31.9]	27.1 [24.7; 31.8]	0.85
Comorbidities, No. (%)					
Cigarette smoking status	193				0.15
Current smoker		22 (11)	13 (12)	9 (11)	
Past-smoker		42 (21)	26 (24)	16 (19)	
Never smoked		84 (44)	52 (47)	32 (39)	
Unknown		45 (23)	19 (17)	26 (31)	
Daily alcohol intake	142				0.002
No		135 (70)	83 (75)	52 (63)	
Yes		7 (4)	7 (4)	0 (0)	
Unknown		50 (26)	20 (18)	30 (37)	
Myocardial infarction	196	16 (8)	8 (7)	8 (9)	0.58
Chronic heart failure	194	24 (12)	13 (12)	11 (13)	0.83
Hypertension	195	129 (66)	75 (68)	54 (64)	0.54
Peripheral vascular disease	194	10 (5)	6 (5)	4 (5)	0.83
Cerebrovascular accident or transient ischemic attack	196	15 (8)	6 (5)	9 (11)	0.18
Presence of sequelae	15	7 (47)	2 (33)	5 (56)	0.40
Hemiplegia	195	3 (2)	0 (0)	3 (4)	0.08
Dementia	196	3 (2)	3 (3)	0 (0)	0.13
COPD	195	17 (9)	11 (10)	6 (7)	0.47
Asthma	195	9 (5)	6 (5)	3 (4)	0.55
Connective tissue disease	195	3 (2)	3 (3)	0 (0)	0.13
Peptic ulcer disease	195	3 (2)	3 (3)	0 (0)	0.13
Chronic liver disease	178	4 (2)	3 (3)	1 (1)	0.59
Diabetes mellitus	173				0.03
Uncomplicated		26 (15)	17 (18)	9 (11)	
End-organ damage		14 (8)	3 (3)	11 (14)	
Type 2 diabetes	194	67 (35)	38 (35)	29 (35)	0.99
Moderate to severe chronic kidney disease	196	22 (11)	6 (5)	16 (19)	0.003
Solid tumor	182				0.67
Localized		11 (12)	11 (11)	10 (13)	
Metastatic		3 (2)	1 (1)	2 (3)	
Leukemia	196	2 (1)	1 (1)	1 (1)	0.85
Lymphoma	195	3 (2)	2 (2)	1 (1)	0.72
AIDS	195	1 (1)	1 (1)	0 (0)	0.56
Arthritis	194	4 (2)	2 (2)	2 (2)	0.77
Mc Cabe score	134				0.35
Non fatal disease		123 (92)	62 (91)	61 (92)	
Ultimately fatal disease		9 (7)	4 (6)	5 (8)	
Rapidly fatal disease		2 (1)	2 (3)	0 (0)	
Medication, n (%)					
Corticosteroids	193	23 (12)	11 (10)	12 (15)	0.01
Inhaled		5 (22)	4 (36)	1 (8)	
Oral low dose		13 (57)	6 (55)	7 (58)	
Oral high dose		5 (22)	1 (9)	4 (33)	
Immuno-modulatory/-suppressant in the last 3 months	190	16 (8)	6 (5)	10 (13)	0.01
ACE inhibitors	196	39 (20)	24 (22)	15 (18)	0.49
Angiotensin II receptor inhibitors	196	32 (16)	21 (19)	11 (13)	0.26
Statins	196	53 (27)	30 (27)	23 (27)	0.99
Proton pump inhibitors	196	36 (18)	23 (21)	13 (15)	0.33

* P values were calculated for comparisons between patients receiving inhaled sedation (for at least 24 hours) and those receiving only intravenous sedation through day 28. Percentages are expressed out of available individuals for each variable, and may not sum to 100% due to rounding. SD: standard deviation; IQR: interquartile

range; COPD: chronic obstructive pulmonary disease; AIDS: acquired immunodeficiency syndrome; ACE: angiotensin converting enzyme.

Table S3. Univariate analysis of the primary endpoint using demographic variables, variables from past medical history, variables from the day before inclusion, and variables from the day of inclusion.

	**Univariate analysis (Kruskal–Wallis or correlation coefficient)**		**Univariate analysis (Zero-inflated negative binomial regression)**			
	**VFD28, median [IQR] or correlation coefficient**	***P***	**Patients with zero VFD28=0, n (%)**	**OR [95%CI] for having zero VFD28, P value**	**VFD28 if VFD28≠0, median [IQR] or correlation coefficient**	**IRR [95%CI] for the number of VFD28 if not zero, *P* value**
**Demographics**						
Gender						
Male	0 [0; 13] (n=147)	0.96	90 (61)	*reference*	17 [8; 21] (n=57)	*reference*
Female	0 [0; 12] (n=44)		26 (59)	0.91 [0.46; 1.82], *P* = 0.79	14 [5; 20] (n=18)	0.91 [0.46; 1.82], *P* = 0.41
Age, years	*Rho* = -0.2866 (n=193)	10^{-4}	117 (61)	1.06 [1.02; 1.09], *P* = 0.001	*Rho* = -0.1293 (n=76)	1.01 [0.99; 1.03], *P* = 0.19
Body mass index, kg/m²	*Rho* = -0.0141 (n=190)	0.85	114 (60)	0.99 [0.94; 1.04], *P* = 0.71	*Rho* = -0.0238 (n=76)	0.99 [0.97; 1.02], *P* = 0.92
**Medical History**						
Smoking status						
Current smoker	0 [0; 19] (n=22)		13 (59)	*reference*	20 [16; 22] (n=9)	*reference*
Past-smoker	0 [0; 4] (n=41)	0.62	28 (68)	1.49 [0.51; 4.35], *P* = 0.47	15 [7; 18] (n=13)	1.34 [0.79; 2.27], *P* = 0.27
Never smoked	0 [0; 14] (n=83)		50 (60)	1.05 [0.40; 2.73], *P* = 0.92	18 [12; 23] (n=33)	1.10 [0.70; 1.73], *P* = 0.68
Unknown	0 [0; 7] (n=44)		26 (55)	0.99 [0.35; 2.81], *P* = 0.99	13 [5; 17] (n=18)	1.61 [0.98; 2.65], *P* = 0.06
Daily alcohol intake						
No	0 [0; 14] (n=134)	0.37	83 (62)	*reference*	18 [12; 23] (n=51)	*reference*
Yes	0 [0; 0] (n=7)		6 (86)	3.67 [0.43; 31.50], *P* = 0.24	12 [12; 12] (n=1)	1.38 [0.40; 4.90], *P* = 0.61
Unknown	0 [0; 6] (n=48)		28 (58)	0.85 [0.43; 2.29], *P* = 0.64	11 [4; 18] (n=20)	1.51 [1.08; 2.10], *P* = 0.02
Arterial hypertension						
No	3 [0; 15] (n=65)	0.02	31 (48)	*reference*	15 [7; 22] (n=34)	*reference*
Yes	0 [0; 8] (n=127)		85 (67)	2.23 [1.21; 4.14], *P* = 0.01	17 [8; 20] (n=42)	0.95 [0.71; 1.28], *P* = 0.75
Myocardial infarction						
No	0 [0; 13] (n=177)	0.38	106 (60)	*reference*	16 [8; 21] (n=71)	*reference*
Yes	0 [0; 2] (n=16)		11 (69)	1.46 [0.49; 4.42], *P* = 0.50	14 [2; 18] (n=5)	1.26 [0.69; 2.29], *P* = 0.46
Chronic heart failure						
No	0 [0; 13] (n=167)	0.20	98 (59)	*reference*	16 [8; 21] (n=69)	*reference*
Yes	0 [0; 4] (n=24)		17 (71)	1.71 [0.67; 4.35], *P* = 0.26	14 [6; 18] (n=7)	1.17 [0.70; 1.95], *P* = 0.54
Peripheral vascular disease		0.34				
No	0 [0; 13] (n=181)		110 (61)	*reference*	16 [8; 20] (n=71)	*reference*
Yes	0 [0; 1] (n=10)		7 (70)	1.46 [0.36; 5.93], *P* = 0.60	5 [1; 16] (n=3)	2.08 [0.92; 4.71], *P* = 0.08
Cerebrovascular accident or transient ischemic attack		0.20				
No	0 [0; 13] (n=178)		106 (60)	*reference*	16 [8; 21] (n=72)	*reference*
Yes	0 [0; 2] (n=15)		11 (73)	1.86 [0.57; 6.05], *P* = 0.31	10 [2; 19] (n=4)	1.48 [0.76; 2.92], *P* = 0.26
Dementia		0.79				
No	0 [0; 13] (n=190)		115 (61)	*reference*	16 [8; 21] (n=75)	*reference*

Yes	0 [0; 2] (n=3)		2 (67)	1.30 [0.12; 14.73], *P* = 0.83	14 [14; 14] (n=1)	1.06 [0.29; 3.88], *P* = 0.93
Hemiplegia						
No	0 [0; 13] (n=189)	0.48	115 (61)	*reference*	16 [8; 21] (n=74)	*reference*
Yes	2 [0; 20] (n=3)		1 (33)	0.20 [0.03; 3.74], *P* = 0.36	11 [2; 20] (n=2)	1.36 [0.53; 3.51], *P* = 0.52
COPD						
No	0 [0; 13] (n=175)	0.18	103 (59)	*reference*	16 [8; 21] (n=72)	*reference*
Yes	0 [0; 0] (n=17)		13 (76)	2.27 [0.71; 7.29], *P* = 0.17	18 [10; 21] (n=4)	0.97 [0.50; 1.87], *P* = 0.93
Asthma						
No	0 [0; 13] (n=183)	0.49	111 (61)	*reference*	16 [8; 21] (n=72)	*reference*
Yes	0 [0; 3] (n=9)		6 (67)	1.27 [0.30; 5.31], *P* = 0.75	5 [3; 17] (n=3)	1.84 [0.83; 4.10], *P* = 0.14
Connective tissue disease		0.84				
No	0 [0; 12] (n=189)		115 (61)	*reference*	16 [8; 20] (n=74)	*reference*
Yes	0 [0; 16] (n=3)		2 (67)	1.28 [0.11; 14.57], *P* = 0.84	16 [16; 16] (n=1)	0.92 [0.25; 3.34], *P* = 0.90
Peptic ulcer disease						
No	0 [0; 13] (n=189)	0.18	113 (60)	*reference*	16 [8; 21] (n=76)	*reference*
Yes	0 [0; 0] (n=3)		3 (2)	*NR*	(n=0)	*NR*
Chronic liver disease						
None	0 [0; 13] (n=171)	0.29	101 (59)	*reference*	16 [8; 20] (n=70)	*reference*
Mild	0 [0; 0] (n=3)		3 (100)	*NR*	(n=0)	*NR*
Moderate/severe	0 [0; 0] (n=1)		1 (100)	*NR*	(n=0)	*NR*
Type 1 diabetes						
None	0 [0; 14] (n=131)	0.36	73 (56)	*reference*	16 [8; 20] (n=58)	*reference*
Uncomplicated	0 [0; 12] (n=25)		17 (68)	1.70 [0.68; 4.22], *P* = 0.25	19 [15; 23] (n=8)	0.81 [0.50; 1.31], *P* = 0.38
End-organ damage	0 [0; 2] (n=14)		10 (71)	1.97 [0.58; 6.66], *P* = 0.27	11 [4; 19] (n=4)	0.92 [0.66; 2.64], *P* = 0.43
Type 2 diabetes						
No	0 [0; 14] (n=126)	0.30	72 (57)	*reference*	16 [8; 22] (n=52)	*reference*
Yes	0 [0; 8] (n=65)		42 (65)	1.28 [0.69; 2.39], *P* = 0.44	13 [7; 19] (n=23)	1.13 [0.82; 1.55], *P* = 0.47
Chronic kidney disease		0.09				
No	0 [0; 13] (n=171)		100 (58)	*reference*	16 [8; 21] (n=71)	*reference*
Yes	0 [0; 0] (n=22)		17 (77)	2.41 [0.85; 6.89], *P* = 0.10	17 [8; 18] (n=5)	1.10 [0.60; 1.99], *P* = 0.76
Solid tumor						
None	0 [0; 13] (n=158)	0.98	93 (59)	*reference*	16 [8; 20] (n=62)	*reference*
Localized	0 [0; 5] (n=21)		12 (57)	0.88 [0.35; 2.23], *P* = 0.79	6 [4; 20] (n=9)	1.23 [0.77; 1.97], *P* = 0.39
Metastatic	0 [0; 28] (n=3)		2 (67)	1.34 [0.12; 15.18], *P* = 0.81	28 [28; 28] (n=1)	0.53 [0.15; 1.86], *P* = 0.32
Leukemia						
No	0 [0; 13] (n=191)	0.27	115 (60)	*reference*	16 [8; 21] (n=76)	*reference*
Yes	0 [0; 0] (n=2)		2 (100)	*NR*	(n=0)	*NR*
Lymphoma						
No	0 [0; 13] (n=189)	0.18	113 (60)	*reference*	16 [8; 21] (n=76)	*reference*
Yes	0 [0; 0] (n=3)		3 (100)	*NR*	(n=0)	*NR*
AIDS						
No	0 [0; 13] (n=191)	0.44	115 (60)	*reference*	16 [8; 21] (n=76)	*reference*
Yes	0 [0; 0] (n=1)		1 (100)	*NR*	(n=0)	*NR*
Arthritis						
No	0 [0; 12] (n=187)	0.52	114 (61)	*reference*	16 [8; 21] (n=73)	*reference*
Yes	9 [0; 19] (n=4)		2 (50)	0.64 [0.09; 4.71], *P* = 0.64	19 [18; 20] (n=2)	0.77 [0.31; 1.92], *P* = 0.57
Other comorbidity						
No	2 [0; 18] (n=94)	0.002	45 (48)	*reference*	17 [8; 21] (n=49)	*reference*
Yes	0 [0; 12] (n=97)		71 (73)	2.97 [1.63; 5.47], $P < 10^{-4}$	15 [12; 20] (n=26)	1.02 [0.74; 1.39], *P* = 0.92

Mc Cabe score		0.19				
Nonfatal disease	0 [0; 17] (n=120)		61 (51)	*reference*	17 [7; 21] (n=59)	*reference*
Ultimately fatal disease	0 [0; 0] (n=9)		7 (78)	*NR*	17 [5; 28] (n=2)	*NR*
Rapidly fatal disease	0 [0; 0] (n=2)		2 (100)	*NR*	(n=0)	*NR*
Corticosteroid therapy		0.91				
No	0 [0; 13] (n=153)		91 (59)	*reference*	16 [8; 21] (n=62)	*reference*
Yes	0 [0; 13] (n=23)		14 (61)	1.06 [0.43; 2.61], *P* = 0.90	17 [8; 19] (n=9)	1.06 [0.67; 1.67], *P* = 0.82
Unknown	0 [0; 5] (n=14)		9 (64)	1.22 [0.39; 3.85], *P* = 0.73	14 [15; 21] (n=5)	1.08 [0.60; 1.97], *P* = 0.78
Immunosuppressant therapy						
No	0 [0; 13] (n=152)	0.50	89 (59)	*reference*	16 [8; 20] (n=63)	*reference*
Yes	0 [0; 19] (n=16)		9 (56)	0.91 [0.88; 2.59], *P* = 0.87	19 [16; 25] (n=7)	0.77 [0.47; 1.26], *P* = 0.30
Unknown	0 [0; 2] (n=19)		13 (68)	1.52 [0.55; 4.25], *P* = 0.42	10 [2; 21] (n=6)	1.25 [0.72; 2.16], *P* = 0.44
Treatment with ACE inhibitors		0.33				
No	0 [0; 12] (n=155)		97 (63)	*reference*	16 [8; 21] (n=58)	*reference*
Yes	0 [0; 16] (n=38)		20 (53)	0.66 [0.32; 1.36], *P* = 0.26	17 [6; 20] (n=18)	1.05 [0.74; 1.49], *P* = 0.78
Treatment with angiotensin II receptor inhibitors		0.60				
No	0 [0; 13] (n=162)		96 (59)	*reference*	16 [7; 20] (n=66)	*reference*
Yes	0 [0; 13] (n=31)		21 (68)	1.45 [0.64; 3.28], *P* = 0.37	19 [13; 25] (n=10)	0.80 [0.52; 1.22], *P* = 0.29
Treatment with statins		0.28				
No	0 [0; 13] (n=140)		82 (59)	*reference*	16 [8; 21] (n=58)	*reference*
Yes	0 [0; 5] (n=53)		35 (66)	1.37 [0.71; 2.66], *P* = 0.35	17 [5; 20] (n=18)	1.11 [0.79; 1.57], *P*=0.55
Treatment with proton pump inhibitors		0.84				
No	0 [0; 13] (n=157)		96 (61)	*reference*	16 [8; 20] (n=61)	*reference*
Yes	0 [0; 12] (n=36)		21 (58)	0.89 [0.42; 1.86], *P* = 0.75	16 [5; 21] (n=15)	1.07 [0.74; 1.55], *P* = 0.73
**Data from day 0 (baseline)**						
Body temperature, °C	*Rho* = 0.0014 (n=111)	0.98	75 (68)	0.88 [0.65; 1.19], *P* = 0.39	*Rho* = 0.0014 (n=75)	1.06 [0.90; 1.25], *P* = 0.48
Heart rate, bpm	*Rho* = -0.0354 (n=190)	0.63	115 (61)	1.00 [0.99; 1.02], *P* = 0.66	*Rho* = -0.1351 (n=75)	1.00 [1.00; 1.01], *P* = 0.26
Mean arterial blood pressure, mmHg	*Rho* = 0.0284 (n=187)	0.70	115 (61)	1.00 [0.98; 1.02], *P* = 0.94	*Rho* = 0.1186 (n=72)	1.00 [0.99; 1.01], *P* = 0.44
Supraventricular tachycardia		0.01				
No	0 [0; 14] (n=178)		103 (58)	*reference*	16 [8; 21] (n=75)	*reference*
Yes	0 [0; 0] (n=10)		10 (100)	*NR*	(n=0)	*NR*
Atrial fibrillation						
No	0 [0; 14] (n=176)	0.02	101 (57)	*reference*	16 [8; 21] (n=75)	*reference*
Yes	0 [0; 0] (n=8)		8 (100)	*NR*	(n=0)	*NR*
Need for vasopressor support		0.001				
No	7 [0; 18] (n=40)		15 (37)	*reference*	17 [11; 20] (n=25)	*reference*
Yes	0 [0; 8] (n=150)		99 (66)	3.25 [1.57; 6.75], *P* = 0.002	15 [7; 21] (n=51)	1.06 [0.78; 1.45], *P* = 0.70
Urine output, n (%)						
>0.5 mL/kg/h	0 [0; 15] (n=134)	0.18	75 (56)	*reference*	17 [7; 22] (n=59)	*reference*
<0.5 ml/kg/h for 6-12 h	0 [0; 12] (n=15)		9 (60)	1.17 [0.39; 3.51], *P* = 0.77	13 [12; 14] (n=6)	1.19 [0.68; 2.08], *P* = 0.55

<0.5 ml/kg/h for ≥12 h	0 [0; 0] (n=20)		16 (75)	3.16 [1.00; 9.94], *P* = 0.05	15 [9; 18] (n=4)	1.16 [0.59; 2.27], *P* = 0.67
<0.3 ml/kg/h for ≥24 h	0 [0; 0] (n=9)		7 (78)	2.77 [0.55; 13.87], *P* = 0.21	24 [22; 25] (n=2)	0.64 [0.26; 1.57], *P* = 0.32
Need for renal replacement therapy		0.72				
No	0 [0; 13] (n=184)		112 (61)	*reference*	17 [8; 21] (n=72)	*reference*
Yes	0 [0; 10] (n=8)		5 (63)	1.06 [0.24; 4.62], *P* = 0.94	13 [7; 13] (n=3)	1.38 [0.63; 3.00], *P* = 0.43
Diabetes insipidus		0.09				
No	0 [0; 0] (n=136)		75 (55)	*reference*	17 [7; 21] (n=61)	*reference*
Suspected	0 [0; 0] (n=4)		4 (100)	*NR*	(n=0)	*NR*
Confirmed	0 [0; 0] (n=3)		3 (100)	*NR*	(n=0)	*NR*
Under intravenous sedation with		0.87				
Propofol	0 [0; 15] (n=114)		68 (60)	*reference*	18 [8; 21] (n=46)	*reference*
Midazolam	0 [0; 10] (n=36)		21 (58)	0.94 [0.44; 2.01], *P* = 0.87	13 [5; 16] (n=15)	1.40 [0.94; 2.10], *P* = 0.09
Other	1 [0; 2] (n=2)		1 (50)	0.21 [0.00; 343.78], *P* = 0.68	2 [2; 2] (n=1)	11.92 [1.17; 120.90], *P* = 0.04
Receiving a second sedative agent		0.78				
No	0 [0; 16] (n=67)		41 (61)	*reference*	18 [12; 24] (n=26)	*reference*
Yes	0 [0; 12] (n=124)		74 (52)	0.93 [0.51; 1.72], *P* = 0.83	14 [6; 20] (n=50)	1.23 [0.91; 1.67], *P* = 0.17
Highest Richmond Agitation Sedation Scale observed	*Rho* = -0.0804 (n=183)	0.28	112 (61)	1.12 [0.92; 1.36], *P* = 0.27	*Rho* = 0.0838 (n=71)	0.99 [0.89; 1.09], *P* = 0.82
Lowest Richmond Agitation Sedation Scale observed	*Rho* = -0.0667 (n=182)	0.28	112 (62)	1.11 [0.90; 1.38], *P* = 0.32	*Rho* = 0.0780 (n=70)	1.03 [0.91; 1.16], *P* = 0.67
Highest Behavioral Pain Scale observed	*Rho* = 0.0597 (n=105)	0.54	63 (60)	1.08 [0.74; 1.61], *P* = 0.67	*Rho* = -0.0287 (n=42)	0.95 [0.75; 1.21], *P* = 0.70
Lowest Behavioral Pain Scale observed	*Rho* = -0.0317 (n=105)	0.75	63 (60)	0.98 [0.15; 6.23], *P* = 0.99	*Rho* = -0.1755 (n=42)	1.77 [0.71; 4.44], *P* = 0.22
Bispectral index available		0.05				
No	0 [0; 12] (n=157)		96 (61)	*reference*	15 [6; 20] (n=61)	*reference*
Yes	11 [0; 18] (n=25)		11 (44)	0.50 [0.21; 1.18], *P* = 0.11	18 [14; 22] (n=14)	0.81 [0.56; 1.18], *P* = 0.27
Highest bispectral index value	*Rho* = 0.2195 (n=25)	0.29	11 (44)	0.95 [0.87; 1.04], *P* = 0.31	*Rho* = 0.1062 (n=14)	0.99 [0.97; 1.02], *P* = 0.62
Lowest bispectral index value	*Rho* = 0.0280 (n=25)	0.89	11 (44)	0.92 [0.77; 1.11], *P*=0.39	*Rho* = -0.1631 (n=14)	1.05 [1.01; 1.09], *P*=0.01
Richmond Agitation Sedation Scale documented by bedside nurse		0.78				
No	0 [0; 10] (n=28)		15 (54)	*reference*	11 [2; 14] (n=13)	*reference*
Yes	0 [0; 16] (n=152)		89 (59)	1.24 [0.55; 2.80], *P* = 0.60	18 [8; 21] (n=63)	0.65 [0.44; 0.96], *P* = 0.03
Pain assessment documented by bedside nurse		0.31				
No	0 [0; 13] (n=81)		43 (53)	*reference*	14 [6; 20] (n=38)	*reference*
Yes	0 [0; 11] (n=100)		63 (63)	1.51 [0.83; 2.75], *P* = 0.18	17 [8; 21] (n=37)	0.90 [0.67; 1.21], *P* = 0.49
CAM-ICU assessment documented by bedside nurse		0.40				

No	0 [0; 12] (n=133)		78 (59)	*reference*	14 [5; 20] (n=55)	*reference*
Yes	0 [0; 18] (n=33)		19 (58)	0.96 [0.44; 2.09], *P* = 0.05	20 [16; 24] (n=14)	0.69 [0.48; 1.00], *P* = 0.92
Spontaneous awakening trial performed		0.25				
No	0 [0; 14] (n=178)		103 (58)	*reference*	16 [8; 21] (n=75)	*reference*
Yes	11 [0; 0] (n=2)		2 (100)	*NR*	(n=0)	*NR*
Ventilation mode		0.09				
Volume-controlled	0 [0; 13] (n=85)		51 (60)	*reference*	16 [8; 22] (n=34)	*reference*
Pressure-controlled	0 [0; 13] (n=55)		32 (58)	0.93 [0.46; 1.85], *P* = 0.83	15 [8; 20] (n=23)	1.07 [0.76; 1.52], *P* = 0.68
Pressure-support	0 [0; 0] (n=14)		13 (93)	8.67 [1.09; 69.90], *P* = 0.04	27 [27; 27] (n=1)	0.55 [0.16; 1.91], *P* = 0.35
Other	1 [0; 18] (n=36)		18 (50)	0.6 [0.30; 1.46], *P* = 0.31	18 [5; 20] (n=18)	1.00 [0.69; 1.45], *P* = 0.98
Tidal volume, mL/kg PBW	*Rho* = -0.1220 (n=187)	0.10	112 (60)	1.23 [0.98; 1.57], *P* = 0.08	*Rho* = -0.0295 (n=75)	1.02 [0.91; 1.14], *P* = 0.80
Dynamic inspiratory plateau pressure, cmH_2O	*Rho* = -0.1472 (n=122)	0.11	74 (61)	1.03 [0.96; 1.11], *P* = 0.40	*Rho* = -0.3307 (n=48)	1.05 [0.99; 1.10], *P* = 0.07
Compliance of the respiratory system, mL/cmH_2O	*Rho* = 0.0531 (n=122)	0.56	74 (61)	1.01 [0.99; 1.03], *P* = 0.50	*Rho* = 0.4156 (n=48)	0.98 [0.97; 0.99], *P* = 0.03
Airway resistance, $cmH_2O/L/s$	*Rho* = 0.1681 (n=67)	0.17	41 (61)	0.96 [0.92; 0.99], *P* = 0.04	*Rho* = 0.2104 (n=26)	0.99 [0.98; 1.01], *P*=0.33
Driving pressure, cmH_2O	*Rho* = -0.1427 (n=122)	0.12	74 (61)	1.02 [0.95; 1.11], *P* = 0.55	*Rho* = -0.3787 (n=48)	1.07 [1.02; 1.12], *P* = 0.005
Receiving adjunctive therapies for ARDS		0.36				
No	0 [0; 15] (n=74)		42 (58)	*reference*	18 [7; 20] (n=32)	*reference*
Yes	0 [0; 12] (n=116)		73 (63)	1.30 [0.71; 2.34], *P* = 0.40	16 [8; 21] (n=43)	1.03 [0.77; 1.39], *P* = 0.83
Receiving neuromuscular blockade		0.12				
No	0 [0; 5] (n=51)		36 (71)	*reference*	16 [5; 20] (n=15)	*reference*
Yes	0 [0; 13] (n=63)		36 (57)	0.56 [0.25; 1.22], *P* = 0.14	16 [11; 22] (n=27)	0.90 [0.60; 1.35], *P* = 0.62
Receiving inhaled nitric oxide		0.09				
No	0 [0; 12] (n=109)		66 (61)	*reference*	16 [8; 21] (n=43)	*reference*
Yes	0 [0; 0] (n=5)		5 (100)	*NR*	(n=0)	*NR*
Receiving prone position		0.66				
No	0 [0; 10] (n=52)		34 (65)	*reference*	17 [8; 21] (n=18)	*reference*
Yes	0 [0; 12] (n=62)		37 (60)	0.78 [0.36; 1.68], *P* = 0.53	13 [8; 20] (n=25)	1.11 [0.74; 1.64], *P* = 0.62
Receiving recruitment maneuver(s)		0.09				
No	0 [0; 5] (n=78)		51 (65)	*reference*	12 [5; 19] (n=27)	*reference*
Yes	0 [0; 18] (n=36)		20 (56)	0.67 [0.30; 1.49], *P* = 0.32	19 [15; 23] (n=16)	0.64 [0.44; 0.93], *P* = 0.02
Receiving extracorporeal membrane oxygenation		0.02				
No	0 [0; 13] (n=103)		61 (59)	*reference*	16 [8; 21] (n=42)	*reference*
Yes	0 [0; 0] (n=9)		9 (100)	*NR*	(n=0)	*NR*
Receiving corticosteroid therapy		0.86				
No	0 [0; 11] (n=85)		50 (59)	*reference*	13 [5; 20] (n=35)	*reference*

Yes	0 [0; 15] (n=107)		66 (62)	1.13 [0.63; 2.03], *P* = 0.67	18 [12; 22] (n=41)	0.77 [0.58; 1.03], *P* = 0.08
Receiving antibiotic therapy		0.46				
No	0 [0; 12] (n=33)		18 (55)	*reference*	15 [5; 20] (n=15)	*reference*
Yes	0 [0; 13] (n=159)		99 (62)	1.38 [0.64; 2.94], *P* = 0.41	17 [8; 21] (n=60)	0.94 [0.65; 1.36], *P* = 0.75
Arterial pH	*Rho* = 0.1164 (n=189)	0.11	114 (60)	0.04 [0.01; 1.57], *P* = 0.09	*Rho* = 0.1707 (n=75)	0.29 [0.05; 1.78], *P* = 0.18
PaO_2/FiO_2, mmHg	*Rho* = 0.2278 (n=181)	0.00 2	109 (60)	0.99 [0.99; 0.99], *P* = 0.03	*Rho* = 0.3304 (n=72)	0.99 [0.99; 0.99], *P* = 0.01
$PaCO_2$, mmHg	*Rho* = -0.1303 (n=189)	0.07	114 (60)	1.01 [0.99; 1.04], *P* = 0.33	*Rho* = -0.3216 (n=75)	1.02 [1.01; 1.04], *P* = 0.01
Serum lactate, mmol/L	*Rho* = -0.1338 (n=165)	0.09	114 (69)	1.43 [0.88; 2.32], *P* = 0.15	*Rho* = 0.0196 (n=65)	1.06 [0.79; 1.43], *P* = 0.68
Serum creatinine, μmol/L	*Rho* = -0.2264 (n=174)	0.00 2	106 (61)	1.01 [1.00; 1.02], *P* = 0.004	*Rho* = -0.0931 (n=68)	1.00 [1.00; 1.00], *P* = 0.74

* *P* values were calculated for comparisons between patients receiving inhaled sedation (for at least 24 hours) and those receiving only intravenous sedation through day 28. VFD28: ventilator-free days through day 28; OR: odds ratio; IRR: incident rate ratio; CI: confidence interval; ACE: angiotensin-converting enzyme; PaO_2: partial pressure of oxygen in arterial blood; FiO_2: fraction of inspired oxygen; $PaCO_2$: partial pressure of carbon dioxide in arterial blood; CAM-ICU: confusion assessment method in the intensive care unit; PWB: predicted body weight; ARDS: acute respiratory distress syndrome.

Table S4. Baseline variables integrated in the propensity score that was used to predict probability of receiving inhaled sedation or not, and derived from the fitted regression model (logistic regression).

	**Coefficient**	**Standard Error**	**z**	***P*>|z|**	**95% Confidence Interval**
Richmond Agitation Sedation Scale documented by bedside nurse	0.30	0.47	0.64	0.53	[-0.63; 1.23]
CAM-ICU assessment documented by bedside nurse	1.86	0.62	2.99	0.003	[0.64; 3.07]
Receiving a second sedative agent	-0.39	0.41	-0.95	0.34	[-1.18; 0.41]
Tidal volume (mL/kg PBW)	0.16	0.13	1.27	0.21	[-0.09; 0.41]
Receiving COVID-19 "specific" therapy	-0.29	0.36	-0.80	0.43	[-1.00; 0.42]

CAM-ICU: confusion assessment method in the intensive care unit; PBW: predicted body weight; COVID-19: coronavirus disease 2019.

Table S5. Selected baseline characteristics (demographics, medical history, clinical and biological data) according to treatment assignment (inhaled versus intravenous sedation) in propensity-matched patients.

**Variable**	**No. of Available Individuals**	**Inhaled Sedation Group (n = 43)**	**IV Sedation Group (n = 29)**	***P* Value***
Demographics				
Age, mean ± SD	72	68 ± 11	67 ± 9	0.95
Men, No. (%)	70	31 (74)	24 (86)	0.23
Body mass index, median (IQR), kg/m^{-2}	72	30 [28; 34]	27 [26; 31]	0.05
Comorbidities, No. (%)				
Cigarette smoking status	72			0.75
Current smoker		6 (14)	3 (10)	
Past-smoker		6 (14)	5 (17)	
Never smoked		21 (50)	12 (41)	
Unknown		9 (21)	9 (31)	

Daily alcohol intake	71			0.40
No		29 (69)	19 (66)	
Yes		2 (5)	0 (0)	
Unknown		11 (26)	10 (34)	
Myocardial infarction	72	2 (5)	3 (10)	0.35
Chronic heart failure	72	6 (14)	4 (14)	0.99
Hypertension	72	33 (77)	21 (72)	0.68
COPD	72	4 (9)	3 (10)	0.88
Asthma	71	3 (7)	1 (4)	0.54
Chronic liver disease	62	2 (6)	1 (4)	0.70
Moderate to severe chronic kidney disease	72	3 (7)	4 (14)	0.34
Diabetes mellitus	60			0.18
Uncomplicated		9 (27)	4 (15)	
End-organ damage		0 (0)	2 (8)	
Type 2 diabetes	70	17 (40)	11 (39)	0.92
Solid tumor	66			0.51
Localized		4 (10)	4 (15)	
Metastatic		0 (0)	0 (0)	
Mc Cabe score	49			0.36
Non fatal disease		27 (90)	19 (100)	
Ultimately fatal disease		2 (7)	0 (0)	
Rapidly fatal disease		1 (3)	0 (0)	
Long-term medication, No (%)				
Corticosteroids	72	5 (12)	2 (7)	0.73
Immuno-modulatory/-suppressant in the last 3 months	71	4 (9)	2 (7)	0.94
Clinical data				
Temperature (°c)	72	37.1 [36.6; 37.8]	37.2 [36.7; 37.8]	0.85
Heart rate (/min)	72	79 [65; 100]	77 [70; 99]	0.87
Mean blood pressure (mmHg)	72	72 [65; 86]	77 [71; 91]	0.10
Supraventricular tachycardia	72	1 (2)	2 (7)	0.34
Atrial fibrillation	70	2 (5)	0 (0)	0.24
Mechanical ventilation data				
Ventilation mode	72			0.002
Volume-controlled ventilation		26 (60)	9 (31)	
Pressure-controlled ventilation		15 (35)	9 (31)	
Pressure-support ventilation		2 (5)	5 (17)	
Other		0 (0)	6 (21)	
Dynamic plateau pressure (cmH_2O)	47	25 [22; 27]	26 [23; 30]	0.30
PEEP (cmH_2O)	72	12 [10; 14]	12 [10; 13]	0.08
Expired tidal volume (mL)	72	440 [375; 480]	451 [419; 507]	0.23
Tidal volume (mL/kg PBW)	72	6.7 [5.9; 7.8]	7.0 [6.2; 7.5]	0.81
Compliance of the respiratory system (mL/cmH_2O)	47	39 [30; 50]	34 [27; 39]	0.09
Driving pressure (cmH_2O)	47	11 [9; 13]	14 [12; 17]	0.04
Sedation				
Receiving sedation	72	43 (100)	29 (100)	-
If intravenous sedation	72	23 (53)	28 (97)	10^{-4}
Sedation with propofol		16 (70)	21 (75)	
Sedation with midazolam		7 (30)	7 (25)	
If inhaled sedation	72	20 (47)	0 (0)	10^{-4}
Sedation with Sevoflurane		13 (65)	0 (0)	
Sedation with Isoflurane		7 (35)	0 (0)	
Use of Sedaconda-ACD-S		20 (100)	0 (0)	
Use of a second sedative agent	72	17 (40)	19 (66)	0.03
Treatments				
Receiving vasopressors, n (%)	71	30 (71)	23 (79)	0.45
Renal replacement therapy	72	2 (5)	1 (3)	0.80
Receiving antibiotics	71	33 (79)	25 (86)	0.41
Tocilizumab	72	1 (11)	0 (0)	0.75
Steroid therapy	71	29 (69)	15 (51)	0.14
Biological data				

PaO_2/FiO_2 (mmHg)	71	121 [91; 155]	128 [85; 174]	0.56
$PaCO_2$ (mmHg)	72	46 [41; 53]	44 [36; 50]	0.20
Serum lactate (mmol/L)	61	1.3 [1.1; 1.7]	1.3 [1.1; 1.5]	0.93
pH	72	7.36 [7.29; 7.40]	7.37 [7.32; 7.43]	0.30
Serum creatinine (µmol/L)	65	86 [70; 114]	81 [59; 107]	0.40

* *P* values were calculated for comparisons between patients receiving inhaled sedation (for at least 24 hours) and those receiving only intravenous sedation through day 28. Percentages are expressed out of available individuals for each variable, and may not sum to 100% due to rounding. Quantitative variables are expressed as median [IQR], qualitative variables as number (%), unless specified otherwise. SD: standard deviation; IQR: interquartile range; COPD: chronic obstructive pulmonary disease; ARDS: acute respiratory distress syndrome; PEEP: positive end expiratory pressure; PBW: predicted body weight; ECMO: extracorporeal membrane oxygenation; PaO_2: partial pressure of oxygen in arterial blood; $PaCO_2$: partial pressure of carbon dioxide in arterial blood.

Table S6. Primary and secondary endpoints according to treatment assignment (inhaled versus intravenous sedation) in propensity-matched patients.

Variable	No. of available individuals	Inhaled Sedation Group (n = 43)	Intravenous Sedation Group (n = 29)	P value*	Relative risk or regression coefficient (95% CI)
Primary endpoint					
VFD28[$]	72	0 [0; 12]	8 [0; 17]	0.06 (OR)<br>0.78 (IRR)	2.56 [0.96; 6.69] (OR)<br>1.05 [0.73; 1.52] (IRR)
Clinical safety endpoints					
Death at day 28, n (%)	71	17 (41)	10 (36)	0.27	0.84 [0.61; 1.15]
ICU-free days through day 28	72	0 [0; 0]	2 [0; 12]	0.01	-0.80 [-1.36; -0.23]
Hospital-free days through day 28	70	0 [0; 0]	0 [0; 6]	0.01	-0.58 [-0.99; -0.17]
Ventilation *(total duration in days)*					
Duration of invasive mechanical ventilation	70	15 [6; 25]	12 [8; 21]	0.74	-0.07 [-0.47; -0.34]
Duration of controlled mechanical ventilation	70	13 [5; 23]	8 [4; 14]	0.11	0.33 [-0.07; 0.74]
Norepinephrine support *(total duration in days)*	71	9 [4; 20]	8 [3; 15]	0.48	0.15 [-0.28; 0.58]
Kidney					
Use of RRT, n (%)		17 (40)	9 (31)	0.43	1.30 [0.68; 2.51]
Total duration of RRT *(days)*	71	12 [3; 20]	10 [7; 22]	0.29	-0.36 [-1.05; 0.32]
Nephrogenic diabetes insipidus	26				
Suspected	58	1 (2)	2 (7)	0.37	0.34 [0.03; 3.55]
Confirmed		1 (2)	1 (3)	0.80	1.35 [0.13; 14.20]
Sedation and NMB *(total duration in days)*					
Inhaled sedation	71	7 [3; 11]	0 [0; 0]	$<10^{-3}$	1.96 [1.67; 2.25]
Intravenous sedation	72	7 [2; 16]	10 [8; 15]	0.02	-0.50 [-0.92; -0.08]
Cisatracurium administration	71	1 [0; 3]	0 [0; 4]	0.99	0.00 [-0.43; 0.43]

Durations are expressed in days since day 0 (study entry). Quantitative variables are expressed as median [interquartile range] and qualitative variables as number (%), unless specified otherwise. *P values were calculated for comparisons between patients receiving inhaled sedation (for at least 24 hours) and those receiving only intravenous sedation through day 28. Effect sizes are expressed as relative risks for categorical variables and regression coefficients for quantitative variables (as expressed for each one-log change in value), with 95% confidence intervals (CI). [$] Zero-inflated negative binomial regression was performed for analysis of the primary endpoint (ventilator-free days through day 28) and results were expressed, with 95% confidence intervals (CI), as the odds ratio (OR) for having zero ventilator-free days through day 28 and the incident rate ratio (IRR) for the number of ventilator-free days when not zero. CI: confidence interval; OR: odds ratio; IRR: incident rate ratio; VFD28: ventilator-free days through day 28; ICU: intensive care unit; RRT: renal replacement therapy; NMB: neuromuscular blockade.

Table S7. Sensitivity analysis of the primary endpoint (ventilator-free days through day 28) when defining groups based on the length of inhaled sedation received (≥2, ≥3, ≥4, ≥5, ≥7 or ≥10 days).

Variable	Univariate analysis (Zero-inflated negative binomial regression)				
	**VFD28, median [IQR]**	**Patients with zero VFD28, no. (%)**	**OR [95%CI] for having zero VFD28**	**VFD28 if VFD28 not zero, median [IQR] or correlation coefficient**	**IRR [95%CI] for the number of VFD28 if not zero**
**Type of Sedation**<br>Intravenous<br>Inhaled ≥ *2 days*	0 [0; 17] (n=96)<br>0 [0; 8] (n=97)	53 (55)<br>64 (66)	*reference*<br>1.57 [0.88; 2.82]<br>*P* = 0.13	18 [8; 22] (n=43)<br>13 [8; 20] (n=33)	*reference*<br>1.14 [0.85; 1.54]<br>*P* = 0.39
**Type of Sedation**<br>Intravenous<br>Inhaled ≥ *3 days*	0 [0; 17] (n=111)<br>0 [0; 8] (n=82)	62 (56)<br>55 (67)	*reference*<br>1.61 [0.89; 2.92]<br>*P* = 0.12	18 [8; 22] (n=49)<br>13 [8; 19] (n=27)	*reference*<br>1.17 [0.87; 1.60]<br>*P* = 0.30
**Type of Sedation**<br>Intravenous<br>Inhaled ≥ *4 days*	0 [0; 17] (n=122)<br>0 [0; 5] (n=71)	66 (54)<br>51 (72)	*reference*<br>2.15 [1.15; 4.04]<br>*P* = 0.02	18 [8; 22] (n=56)<br>12 [7; 16] (n=20)	*reference*<br>1.36 [0.98; 1.90]<br>*P* = 0.07
**Type of Sedation**<br>Intravenous<br>Inhaled ≥ *5 days*	0 [0; 17] (n=134)<br>0 [0; 2] (n=59)	73 (54)<br>44 (75)	*reference*<br>2.44 [1.23; 4.81]<br>*P* = 0.01	18 [8; 22] (n=61)<br>12 [6; 15] (n=15)	*reference*<br>1.43 [0.99; 2.08]<br>*P* = 0.06
**Type of Sedation**<br>Intravenous<br>Inhaled ≥ *7 days*	0 [0; 16] (n=145)<br>0 [0; 1] (n=48)	81 (56)<br>36 (75)	*reference*<br>2.36 [1.13; 4.90]<br>*P* = 0.02	18 [8; 22] (n=64)<br>13 [6; 16] (n=12)	*reference*<br>1.40 [0.94; 2.12]<br>*P* = 0.10
**Type of Sedation**<br>Intravenous<br>Inhaled ≥ *10 days*	0 [0; 14] (n=162)<br>0 [0; 5] (n=31)	94 (58)<br>23 (74)	*reference*<br>2.08 [0.87; 4.90]<br>*P* = 0.10	17 [8; 22] (n=68)<br>13 [7; 16] (n=8)	*reference*<br>1.31 [0.81; 2.14]<br>*P* = 0.27

VFD28: ventilator-free days through day 28; OR: odds ratio; CI: confidence interval; IQR: interquartile range; IRR: incident rate ratio.

Table S8. Subgroup unadjusted analysis of the primary endpoint (ventilator-free days through day 28).

	**OR [95%CI] for having zero VFD28, *P* value**	**P for treatment × subgroup interaction**	**IRR [95%CI] for the number of VFD28 if not zero, P value**	**P for treatment × subgroup interaction**
**Presence of mild ARDS (PaO_2/FiO_2 of 200-299 mmHg) at baseline**				
No (n=176)	1.57 [0.84–2.92], *P*=0.15	0.41	1.10 [0.79–1.52], *P*=0.57	0.73
Yes (n=17)	2.41 [0.30–19.11], *P*=0.41		1.27[0.72–2.27], *P*=0.41	
**Presence of severe ARDS (PaO_2/FiO_2 <100 mmHg) at baseline**				
No (n=147)	1.75 [0.90–3.39], *P*=0.10	0.88	1.11 [0.84–1.48], *P*=0.48	0.38
Yes (n=46)	0.92 [0.22–3.86], *P*=0.91		0.75 [0.24–2.29], *P*=0.61	
**Need for vasopressor support at baseline**				
No (n=40)	6.69 [0.72–12.00], *P*=0.13	0.22	1.42 [0.88–2.29], *P*=0.15	0.26
Yes (n=150)	1.54 [0.78–3.03], *P*=0.22		1.01 [0.70–1.45], *P*=0.97	
**Receiving isoflurane at baseline**				
No (n=183)	1.60 [0.88–2.92], *P*=0.12	0.17	1.19 [0.88–1.60], *P*=0.26	0.99

Yes (n=13)	2.03 [0.53–7.77], *P*=0.30		0.69 [0.33–1.43], *P*=0.32	
**Receiving sevoflurane at baseline**				
No (n=167)	1.72 [0.91–3.25], *P*=0.09	0.29	1.19 [0.84–1.6], *P*=0.32	0.86
Yes (n=29)	1.07 [0.48–2.92], *P*=0.86		0.95 [0.63–1.45], *P*=0.82	
**Intravenous sedative agent at baseline**				
Propofol (n=116)	1.73 [0.89–3.39], *P*=0.11	0.37	1.16 [0.81–1.68], *P*=0.41	0.49
Midazolam (n=36)	0.93 [0.43–2.01], *P*=0.86		1.35 [0.90–2.03], *P*=0.16	
Other (n=2)	0.16 [0.00–1.73], *P*=0.62		11.02 [1.04–109.95], *P*=0.05	
**Receiving inhaled sedation for ≥5 days**				
No (n=134)	1.04 [0.51–2.12], *P*=0.91	0.01	0.99 [0.70–1.40], *P*=0.97	0.09
Yes (n=62)	2.44 [1.23–4.81], *P*=0.01		1.53 [0.99–2.08], *P*=0.06	
**Receiving intravenous sedation for ≥11 days**				
No (n=92)	2.29 [0.96–5.42], *P*=0.06	0.32	1.22 [0.96–1.55], *P*=0.10	0.81
Yes (n=104)	1.55 [0.64–3.71], *P*=0.33		1.16 [0.63–2.14], *P*=0.64	
**Patients admitted in an ICU-like unit**				
No (n=189)	1.65 [0.91–3.00], *P*=0.10	*NA*	1.13 [0.84–1.52], *P*=0.43	*NA*
Yes (n=4)	*NA*		*NA*	

NA: Not applicable (VFD28 = 0 for all patients); VFD28: ventilator-free days through day 28; OR: odds ratio; CI: confidence interval; IQR: interquartile range; IRR: incident rate ratio; ARDS: acute respiratory distress syndrome; PaO_2: partial pressure of oxygen in arterial blood; FiO_2: fraction of inspired oxygen; ICU: intensive care unit.

Table S9. Mechanical ventilation data and adjunctive ARDS therapies received during the first seven days after inclusion.

			Day 1	Day 2	Day 3	Day 4	Day 5	Day 6	Day 7
Ventilation mode	No. of observations available		193	189	185	184	179	172	165
Volume-controlled ventilation	Inhaled Sedation Group	Number of patients — no. (%)	53 (48)	50 (46)	40 (37)	41 (39)	35 (34)	34 (34)	32 (33)
Pressure-controlled ventilation	Inhaled Sedation Group	Number of patients — no. (%)	23 (21)	20 (18)	19 (18)	17 (16)	18 (17)	14 (14)	16 (17)
Pressure-support ventilation	Inhaled Sedation Group	Number of patients — no. (%)	23 (21)	25 (23)	33 (31)	35 (33)	34 (33)	37 (37)	32 (33)
APRV	Inhaled Sedation Group	Number of patients — no. (%)	0 (0)	0 (0)	2 (2)	0 (0)	1 (1)	0 (0)	0 (0)
HFNO	Inhaled Sedation Group	Number of patients — no. (%)	1 (1)	3 (3)	2 (2)	2 (3)	3 (3)	3 (3)	3 (3)
CPAP/NIV	Inhaled Sedation Group	Number of patients — no. (%)	0 (0)	0 (0)	2 (2)	1 (1)	3 (3)	2 (2)	2 (2)
Other	Inhaled Sedation Group	Number of patients — no. (%)	10 (9)	11 (10)	10 (9)	25 (32)	9 (9)	10 (10)	10 (10)
None	Inhaled Sedation Group	Number of patients — no. (%)	0 (0)	0 (0)	0 (0)	0 (0)	0 (0)	0 (0)	1 (1)
Volume-controlled ventilation	Intravenous Sedation Group	Number of patients — no. (%)	25 (30)	22 (28)	21 (27)	17 (22)	17 (22)	14 (19)	15 (22)
Pressure-controlled ventilation	Intravenous Sedation Group	Number of patients — no. (%)	13 (16)	9 (11)	9 (12)	8 (10)	7 (9)	4 (6)	4 (6)
Pressure-support ventilation	Intravenous Sedation Group	Number of patients — no. (%)	13 (16)	15 (19)	14 (18)	22 (28)	20 (26)	22 (31)	18 (26)
APRV	Intravenous Sedation Group	Number of patients — no. (%)	0 (0)	0 (0)	0 (0)	0 (0)	0 (0)	0 (0)	0 (0)
HFNO	Intravenous Sedation Group	Number of patients — no. (%)	0 (0)	1 (1)	1 (1)	0 (0)	1 (1)	1 (1)	1 (1)
CPAP/NIV	Intravenous Sedation Group	Number of patients — no. (%)	0 (0)	0 (0)	0 (0)	0 (0)	1 (1)	0 (0)	2 (3)
Other	Intravenous Sedation Group	Number of patients — no. (%)	32 (39)	33 (41)	30 (39)	9 (8)	28 (37)	28 (39)	27 (39)
None	Intravenous Sedation Group	Number of patients — no. (%)	0 (0)	0 (0)	0 (0)	7 (7)	2 (3)	3 (4)	2 (3)
	P Value*		10^{-4}	10^{-4}	10^{-4}	10^{-4}	10^{-4}	10^{-4}	0.001
Dynamic plateau pressure (cmH_2O)	No. of observations available		123	116	109	105	101	98	90
	Inhaled Sedation Group	Median value [IQR]	26 [21; 28]	26 [22; 28]	26 [24; 29]	27 [23; 29]	26 [23; 30]	27 [23; 30]	27 [21; 30]
	Intravenous Sedation Group	Median value [IQR]	26 [22; 29]	25 [21; 28]	26 [22; 29]	26 [24; 30]	27 [24; 30]	25 [23; 30]	25 [21; 28]
	P Value*		0.69	0.33	0.56	0.70	0.33	0.75	0.51
PEEP (cmH_2O)	No. of observations available		193	187	182	176	173	163	153
	Inhaled Sedation Group	Median value [IQR]	12 [10; 14]	12 [10; 13]	12 [9; 13]	12 [10; 14]	11 [10; 13]	12 [9; 13]	12 [10; 13]
	Intravenous Sedation Group	Median value [IQR]	12 [10; 14]	12 [10; 14]	12 [10; 12]	12 [10; 14]	12 [9; 12]	10 [9; 13]	11 [8; 13]

	P Value*		0.99	0.96	0.38	0.73	0.77	0.66	0.32
Expired tidal volume (mL/kg PBW)	No. of observations available		190	184	180	170	167	157	148
	Inhaled Sedation Group	Median value [IQR]	6.9 [6.0; 8.0]	6.7 [6.1; 7.9]	6.9 [6.3; 8.5]	7.2 [6.2; 8.5]	6.8 [6.1; 7.8]	7.2 [6.3; 8.3]	7.1 [6.3; 8.2]
	Intravenous Sedation Group	Median value [IQR]	6.5 [5.9; 7.4]	6.7 [5.7; 7.3]	6.5 [5.7; 7.4]	6.7 [6.0; 7.8]	6.7 [6.0; 7.5]	6.7 [5.7; 7.8]	6.9 [6.0; 8.0]
	P Value*		0.07	0.06	0.004	0.08	0.55	0.03	0.37
Compliance of the respiratory system (mL/cmH_2O)	No. of observations available		122	115	109	103	100	96	89
	Inhaled Sedation Group	Median value [IQR]	37 [31; 43]	34 [26; 44]	29 [24; 39]	31 [26; 38]	32 [25; 38]	30 [24; 39]	32 [25; 42]
	Intravenous Sedation Group	Median value [IQR]	34 [27; 45]	35 [27; 40]	33 [25; 43]	33 [26; 39]	31 [25; 38]	30 [25; 39]	32 [25; 43]
	P Value*		0.26	0.79	0.25	0.69	0.77	0.63	0.87
Driving pressure (cmH_2O)	No. of observations available		122	54	109	103	100	96	89
	Inhaled Sedation Group	Median value [IQR]	12 [10; 15]	14 [9; 23]	15 [12; 17]	14 [12; 17]	15 [12; 17]	15 [12; 18]	14 [12; 18]
	Intravenous Sedation Group	Median value [IQR]	13 [10; 14]	15 [12; 33]	13 [10; 16]	14 [12; 16]	14 [12; 17]	14 [12; 17]	13 [12; 17]
	P Value*		0.60	0.62	0.13	0.81	0.63	0.50	0.75
Airway resistance (cmH_2O/L/s)	No. of observations available		62	116	53	53	48	47	44
	Inhaled Sedation Group	Median value [IQR]	14 [9; 22]	13 [11; 16]	15 [8; 18]	14 [7; 19]	15 [10; 17]	14 [8; 16]	13 [8; 18]
	Intravenous Sedation Group	Median value [IQR]	11 [7; 36]	13 [10; 15]	14 [9; 38]	14 [8; 28]	11 [10; 21]	14 [9; 17]	14 [8; 17]
	P Value*		0.51	0.27	0.64	0.63	0.45	0.62	0.90
Adjunctive therapies for ARDS	No. of observations available		191	188	186	186	182	175	163
	Inhaled Sedation Group	Number of patients — no. (%)	51 (48)	49 (46)	49 (45)	44 (41)	48 (46)	39 (39)	35 (37)
	Intravenous Sedation Group	Number of patients — no. (%)	60 (71)	53 (65)	48 (62)	49 (62)	43 (56)	43 (58)	40 (58)
	P Value*		10^{-4}	0.007	0.03	0.005	0.18	0.01	0.01
Recruitment maneuvers	No. of observations available		109	100	95	92	90	81	72
	Inhaled Sedation Group	Number of patients — no. (%)	19 (38)	17 (36)	17 (35)	13 (30)	14 (29)	12 (32)	6 (18)
	Intravenous Sedation Group	Number of patients — no. (%)	12 (20)	12 (23)	14 (30)	12 (25)	13 (31)	6 (14)	9 (23)
	P Value*		0.04	0.14	0.56	0.63	0.85	0.06	0.61
Neuromuscular blocking drugs	No. of observations available		110	100	96	89	90	80	74
	Inhaled Sedation Group	Number of patients — no. (%)	32 (64)	28 (60)	28 (58)	23 (55)	22 (46)	17 (46)	15 (44)
	Intravenous Sedation Group	Number of patients — no. (%)	25 (42)	24 (45)	22 (46)	20 (43)	20 (48)	21 (49)	24 (60)
	P Value*		0.02	0.15	0.22	0.25	0.87	0.80	0.17
Inhaled nitric oxide	No. of observations available		108	97	94	91	90	80	73
	Inhaled Sedation Group	Number of patients — no. (%)	6 (12)	10 (22)	11 (23)	9 (20)	8 (17)	11 (24)	9 (27)
	Intravenous Sedation Group	Number of patients — no. (%)	1 (2)	5 (10)	5 (11)	7 (15)	9 (21)	5 (12)	2 (5)

	P Value*		0.03	0.09	0.10	0.49	0.57	0.04	0.01
Prone position	No. of observations available		107	99	95	92	90	81	73
	Inhaled Sedation Group	Number of patients — no. (%)	24 (51)	21 (46)	25 (52)	21 (48)	21 (44)	18 (47)	16 (47)
	Intravenous Sedation Group	Number of patients — no. (%)	44 (73)	31 (58)	31 (66)	27 (56)	21 (50)	23 (53)	23 (59)
	P Value*		0.02	0.20	0.17	0.41	0.55	0.58	0.31
ECMO	No. of observations available		108	95	95	91	89	78	71
	Inhaled Sedation Group	Number of patients — no. (%)	3 (6)	3 (4)	3 (6)	6 (14)	5 (10)	4 (11)	4 (13)
	Intravenous Sedation Group	Number of patients — no. (%)	6 (10)	6 (12)	6 (13)	6 (13)	6 (15)	6 (14)	7 (18)
	P Value*		0.45	0.38	0.28	0.84	0.55	0.74	0.53

* *P* values were calculated for comparisons between patients receiving inhaled sedation (for at least 24 hours) and those receiving only intravenous sedation through day 28. Percentages are expressed out of available individuals for each variable, and may not sum to 100% due to rounding. Quantitative variables are expressed as median [IQR], qualitative variables as number (%), unless specified otherwise. ARDS: acute respiratory distress syndrome; APRV: airway pressure release ventilation; HFNO: high flow nasal oxygenation; CPAP: continuous positive airway pressure; NIV: non-invasive ventilation; PEEP: positive end expiratory pressure; V_T: tidal volume; PBW: predicted body weight; ECMO: extracorporeal membrane oxygenation.

Table S10. Physiological measures of lung function and ventilator settings through day 7 in patients receiving inhaled sedation and those receiving intravenous sedation only.

**Variable**	**Inhaled Sedation Group (n=111)**		**Intravenous Sedation Group (n=85)**		***P* Value for Time × Group Interaction**
**PaO_2/FiO_2, mmHg**	*No. of available individuals*		*No. of available individuals*		
Day 0	108	128 [94; 165]	76	135 [104; 174]	*Reference*
Day 1	99	170 [116; 203]	83	158 [125; 202]	0.94
Day 2	103	155 [118; 195]	81	172 [119; 211]	0.88
Day 3	99	140 [112; 191]	76	157 [126; 215]	0.43
Day 4	97	153 [117; 180]	77	168 [120; 210]	0.20
Day 5	93	145 [106; 200]	75	164 [113; 209]	0.42
Day 6	88	139 [113; 195]	73	142 [117; 196]	0.83
Day 7	87	138 [115; 203]	68	171 [114; 216]	0.16
**$PaCO_2$, mmHg**	*No. of available individuals*		*No. of available individuals*		
Day 0	109	46 [41; 52]	83	44 [38; 51]	*Reference*
Day 1	101	44 [40; 51]	84	44 [40; 48]	0.35
Day 2	104	46 [42; 53]	82	45 [40; 50]	0.25
Day 3	100	47 [42; 55]	79	45 [41; 52]	0.50
Day 4	99	48 [43; 55]	79	45 [40; 51]	0.77
Day 5	96	49 [43; 57]	77	44 [40; 52]	0.04
Day 6	91	50 [42; 59]	74	46 [40; 55]	0.48
Day 7	89	51 [43; 60]	69	47 [42; 55]	0.36
**Dynamic inspiratory plateau pressure, cmH_2O**	*No. of available individuals*		*No. of available individuals*		
Day 0	62	26 [22; 28]	63	25 [22; 30]	*Reference*
Day 1	59	26 [21; 28]	64	26 [22; 29]	0.96
Day 2	55	26 [22; 28]	61	25 [21; 28]	0.03
Day 3	50	26 [24; 29]	59	26 [22; 29]	0.15
Day 4	50	27 [23; 29]	55	26 [24; 30]	0.32
Day 5	46	26 [23; 29]	55	27 [24; 30]	0.95
Day 6	46	27 [23; 30]	52	25 [23; 30]	0.11
Day 7	41	27 [21; 30]	49	25 [21; 28]	0.07

**Compliance of the respiratory system, mL/cmH_2O**	*No. of available individuals*		*No. of available individuals*		
Day 0	62	34 [28; 43]	63	34 [26; 40]	*Reference*
Day 1	58	37 [31; 43]	64	34 [27; 45]	0.78
Day 2	55	34 [26; 44]	61	35 [27; 40]	0.21
Day 3	50	29 [24; 39]	59	33 [25; 43]	0.22
Day 4	49	31 [26; 38]	54	33 [26; 39]	0.44
Day 5	45	32 [25; 38]	55	31 [25; 38]	0.72
Day 6	44	30 [24; 39]	52	31 [25; 39]	0.01
Day 7	40	32 [25; 42]	49	32 [25; 43]	0.34
**Driving pressure, cmH_2O**	*No. of available individuals*		*No. of available individuals*		
Day 0	62	13 [10; 15]	63	12 [10; 16]	*Reference*
Day 1	58	12 [10; 15]	64	13 [10; 14]	0.97
Day 2	55	13 [11; 16]	61	13 [10; 15]	0.09
Day 3	50	15 [12; 17]	59	13 [10; 16]	0.08
Day 4	49	14 [12; 17]	55	14 [12; 16]	0.76
Day 5	45	14 [12; 17]	55	15 [12; 17]	0.81
Day 6	44	15 [12; 18]	52	14 [12; 17]	0.22
Day 7	40	14 [12; 18]	49	13 [12; 17]	0.26
**Airway resistance, cmH_2O/L/s**	*No. of available individuals*		*No. of available individuals*		
Day 0	51	14 [12; 17]	16	15 [11; 27]	*Reference*
Day 1	47	14 [9; 22]	15	11 [7; 36]	0.39
Day 2	43	14 [9; 23]	11	15 [12; 33]	0.49
Day 3	41	15 [8; 18]	12	14 [9; 38]	0.33
Day 4	43	14 [7; 19]	10	14 [8; 28]	0.85
Day 5	36	15 [10; 17]	12	11 [10; 21]	0.41
Day 6	34	14 [8; 16]	13	14 [9; 17]	0.86
Day 7	34	13 [8; 18]	10	14 [8; 17]	0.82
**Tidal volume, mL/kg PBW**	*No. of available individuals*		*No. of available individuals*		
Day 0	108	6.6 [6.0; 7.4]	82	6.4 [5.5; 7.1]	*Reference*
Day 1	109	6.9 [6.0; 8.0]	81	6.5 [5.9; 7.4]	0.95
Day 2	105	6.7 [6.1; 7.9]	79	6.7 [5.7; 7.3]	0.55
Day 3	105	6.9 [6.3; 8.5]	75	6.5 [5.7; 7.4]	0.08
Day 4	100	7.2 [6.2; 8.5]	70	6.7 [6.0; 7.8]	0.61
Day 5	97	6.8 [6.1; 7.8]	70	6.7 [6.0; 7.5]	0.39
Day 6	90	7.2 [6.3; 8.3]	67	6.7 [5.7; 7.8]	0.91
Day 7	84	7.1 [6.3; 8.2]	64	6.9 [6.0; 8.0]	0.85
**Positive end-expiratory pressure, cmH_2O**	*No. of available individuals*		*No. of available individuals*		
Day 0	110	12 [10; 14]	85	12 [10; 14]	*Reference*
Day 1	110	12 [10; 14]	83	12 [10; 14]	0.27
Day 2	106	12 [10; 13]	81	12 [10; 14]	0.52
Day 3	105	12 [9; 13]	77	12 [10; 12]	0.22
Day 4	104	12 [10; 14]	72	12 [10; 14]	0.32
Day 5	101	12 [9; 12]	72	11 [10; 13]	0.26
Day 6	94	12 [9; 13]	69	10 [9; 13]	0.85
Day 7	87	12 [10; 13]	66	11 [8; 13]	0.42
**Fraction of inspired oxygen, %**	*No. of available individuals*		*No. of available individuals*		
Day 0	109	70 [55; 90]	76	70 [52; 80]	*Reference*
Day 1	100	51 [40; 60]	83	53 [40; 70]	0.67
Day 2	103	50 [40; 65]	81	46 [38; 61]	0.82
Day 3	100	51 [45; 61]	76	50 [39; 60]	0.35
Day 4	97	55 [45; 64]	78	50 [35; 60]	0.05
Day 5	93	60 [45; 70]	75	50 [40; 70]	0.31

Day 6	88	55 [45; 65]	73	54 [40; 70]	0.37
Day 7	87	50 [40; 65]	68	50 [35; 66]	0.38
**Respiratory rate, per minute**	*No. of available individuals*		*No. of available individuals*		
Day 0	89	22 [20; 28]	45	22 [18; 25]	*Reference*
Day 1	71	26 [22; 30]	36	24 [20; 27]	0.69
Day 2	62	26 [24; 30]	28	26 [22; 29]	0.87
Day 3	55	28 [24; 30]	28	24 [22; 28]	0.26
Day 4	52	28 [24; 30]	23	27 [24; 29]	0.31
Day 5	47	28 [24; 30]	22	28 [24; 30]	0.79
Day 6	44	28 [26; 30]	16	28 [24; 30]	0.92
Day 7	45	30 [26; 31]	16	26 [24; 29]	0.39

PaO_2: partial pressure of oxygen in arterial blood; FiO_2: fraction of inspired oxygen; $PaCO_2$: partial pressure of carbon dioxide in arterial blood; PBW: predicted body weight.

Table S11. Sedation practices and measures from the ABCDEF bundle implemented during the first seven days after inclusion.

			**Day 1**	**Day 2**	**Day 3**	**Day 4**	**Day 5**	**Day 6**	**Day 7**
Receiving sedation	No. of observations available		195	191	185	187	182	175	166
Receiving sedation	Inhaled Sedation Group	Number of patients — no. (%)	110 (99)	103 (95)	101 (93)	98 (92)	95 (90)	89 (88)	85 (88)
Receiving sedation	Intravenous Sedation Group	Number of patients — no. (%)	82 (98)	81 (99)	74 (93)	69 (86)	69 (90)	65 (88)	63 (91)
Receiving sedation	P Value*		0.41	0.12	0.97	0.24	0.85	0.96	0.45
If intravenous sedation	No. of observations available		192	184	175	167	164	154	148
Sedation with propofol	Inhaled Sedation Group	Number of patients — no. (%)	*43 (70)*	*34 (68)*	*33 (75)*	*32 (71)*	*32 (70)*	*31 (70)*	*26 (63)*
Sedation with midazolam			*15 (25)*	*12 (24)*	*6 (14)*	*6 (13)*	*6 (13)*	*4 (9)*	*6 (15)*
Sedation with dexmedetomidine			*2 (3)*	*2 (4)*	*3 (7)*	*6 (13)*	*7 (15)*	*8 (18)*	*6 (15)*
Sedation with another agent agent			*1 (2)*	*2 (4)*	*2 (5)*	*1 (2)*	*0 (0)*	*1 (2)*	*1 (2)*
Sedation with propofol	Intravenous Sedation Group	Number of patients — no. (%)	*61 (74)*	*60 (75)*	*52 (71)*	*46 (68)*	*42 (62)*	*38 (59)*	*38 (61)*
Sedation with midazolam			*21 (26)*	*19 (24)*	*18 (25)*	*17 (25)*	*17 (25)*	*20 (31)*	*17 (27)*
Sedation with dexmedetomidine			*0 (0)*	*1 (1)*	*2 (3)*	*3 (3)*	*5 (7)*	*4 (6)*	*5 (8)*
Sedation with another agent agent			*0 (0)*	*0 (0)*	*1 (1)*	*2 (3)*	*3 (4)*	*1 (2)*	*1 (2)*
If intravenous sedation	P Value*		10^{-4}	10^{-4}	10^{-4}	10^{-4}	10^{-4}	10^{-4}	10^{-4}
If inhaled sedation	No. of observations available		110	184	189	167	164	154	148
Sedation with Sevoflurane	Inhaled Sedation Group	Number of patients — no. (%)	*49 (45)*	*53 (51)*	*41 (72)*	*29 (55)*	*21 (43)*	*17 (38)*	*16 (36)*
Sedation with Isoflurane			*39 (80)*	*41 (77)*	*16 (28)*	*24 (45)*	*28 (57)*	*28 (62)*	*28 (64)*
Use of Sedaconda-ACD (100 mL)			*10 (20)*	*12 (23)*	*1 (2)*	*43 (81)*	*2 (4)*	*3 (7)*	*2 (5)*
Use of Sedaconda-ACD-S (50 mL)			*45 (92)*	*47 (89)*	*47 (82)*	*2 (4)*	*38 (78)*	*35 (78)*	*35 (80)*
Use of ventilator/vaporizer			*4 (8)*	*6 (11)*	*9 (16)*	*8 (15)*	*9 (18)*	*7 (16)*	*7 (16)*
Sedation with Sevoflurane	Intravenous Sedation Group	Number of patients — no. (%)	*0 (0)*	*0 (0)*	*0 (0)*	*0 (0)*	*0 (0)*	*0 (0)*	*0 (0)*
Sedation with Isoflurane			*0 (0)*	*0 (0)*	*0 (0)*	*0 (0)*	*0 (0)*	*0 (0)*	*0 (0)*
Use of Sedaconda-ACD (100 mL)			*0 (0)*	*0 (0)*	*0 (0)*	*0 (0)*	*0 (0)*	*0 (0)*	*0 (0)*
Use of Sedaconda-ACD-S (50 mL)			*0 (0)*	*0 (0)*	*0 (0)*	*0 (0)*	*0 (0)*	*0 (0)*	*0 (0)*
Use of ventilator/vaporizer			*0 (0)*	*0 (0)*	*0 (0)*	*0 (0)*	*0 (0)*	*0 (0)*	*0 (0)*
If inhaled sedation	P Value*		10^{-4}	10^{-4}	10^{-4}	10^{-4}	10^{-4}	10^{-4}	10^{-4}
Use of a second sedative agent	No. of observations available		188	183	173	165	162	162	146
Use of a second sedative agent	Inhaled Sedation Group	Number of patients — no. (%)	59 (55)	60 (58)	67 (68)	71 (74)	70 (75)	70 (75)	55 (66)
Use of a second sedative agent	Intravenous Sedation Group	Number of patients — no. (%)	66 (83)	70 (88)	65 (88)	59 (86)	54 (72)	54 (78)	49 (78)
Use of a second sedative agent	P Value*		10^{-4}	10^{-4}	0.002	0.07	0.66	0.66	0.13
Maximal RASS observed	No. of observations available		183	174	166	161	160	151	145
Maximal RASS observed	Inhaled Sedation Group	Median value [IQR]	-4 [-5; -3]	-4 [-5; -3]	-4 [-5; -4]	-4 [-5; -4]	-5 [-4; -4]	-4 [-5; -4]	-4 [-5; -4]

	Intravenous Sedation Group	Median value [IQR]	-4 [-5; -4]	4 [-5; -4]	-4 [-5; -4]	-4 [-5; -4]	-5 [-4; -4]	-4 [-5; -4]	-4 [-5; -4]
	P Value*		0.94	0.54	0.44	0.74	0.97	0.15	0.15
Minimal RASS observed	No. of observations available		184	175	169	161	160	150	143
	Inhaled Sedation Group	Median value [IQR]	-4 [-5; -4]	4 [-5; -4]	-5 [-5; -4]	-5 [-5; -4]	-5 [-5; -4]	-5 [-5; -4]	-4 [-5; -3]
	Intravenous Sedation Group	Median value [IQR]	-5 [-5; -4]	-5 [-5; -4]	-4 [-5; -3]	-4 [-5; -3]	-4 [-5; -3]	-4 [-5; -3]	-5 [-5; -4]
	P Value*		10^{-4}	0.04	10^{-4}	10^{-4}	0.01	0.01	0.01
Maximal BPS observed	No. of observations available		106	99	96	95	91	86	83
	Inhaled Sedation Group	Median value [IQR]	3 [3; 3]	3 [3; 3]	3 [3; 3]	3 [3; 4]	3 [3; 4]	3 [3; 3]	3 [3; 5]
	Intravenous Sedation Group	Median value [IQR]	3 [3; 4]	3 [3; 4]	3 [3; 4]	3 [3; 4]	3 [3; 4]	3 [3; 4]	3 [3; 4]
	P Value*		0.51	0.96	0.12	0.57	0.97	0.15	0.36
Minimal BPS observed	No. of observations available		105	99	94	95	91	86	83
	Inhaled Sedation Group	Median value [IQR]	3 [3; 3]	3 [3; 3]	3 [3; 3]	3 [3; 3]	3 [3; 3]	3 [3; 3]	3 [3; 3]
	Intravenous Sedation Group	Median value [IQR]	3 [3; 3]	3 [3; 3]	3 [3; 3]	3 [3; 3]	3 [3; 3]	3 [3; 3]	3 [3; 3]
	P Value*		0.28	0.52	0.23	0.83	0.39	0.90	0.70
BIS monitoring available	No. of observations available		181	171	166	156	149	145	137
	Inhaled Sedation Group	Number of patients — no. (%)	16 (16)	12 (13)	12 (13)	10 (11)	10 (12)	10 (12)	7 (9)
	Intravenous Sedation Group	Number of patients — no. (%)	12 (15)	11 (14)	10 (14)	11 (16)	9 (13)	7 (11)	8 (13)
	P Value*		0.88	0.91	0.88	0.38	0.82	0.84	0.48
Highest BIS value	No. of observations available		28	23	22	20	19	17	15
	Inhaled Sedation Group	Median value [IQR]	55 [47; 59]	58 [52; 64]	54 [42; 64]	57 [46; 66]	58 [55; 72]	58 [44; 62]	60 [43; 70]
	Intravenous Sedation Group	Median value [IQR]	62 [50; 68]	53 [43; 65]	55 [43; 66]	63 [51; 74]	66 [58; 88]	61 [54; 77]	61 [44; 71]
	P Value*		0.13	0.28	0.84	0.55	0.33	0.59	0.91
Lowest BIS value	No. of observations available		28	23	22	20	19	17	15
	Inhaled Sedation Group	Median value [IQR]	40 [38; 42]	41 [34; 43]	39 [30; 42]	41 [18; 42]	36 [16; 43]	30 [18; 39]	38 [15; 42]
	Intravenous Sedation Group	Median value [IQR]	41 [31; 46]	38 [30; 44]	40 [28; 45]	36 [31; 44]	40 [31; 42]	35 [27; 44]	30 [17; 40]
	P Value*		0.66	0.69	0.53	0.73	0.49	0.49	0.52
CAM-ICU<br>*Positive*<br>*Negative*<br>*Unable to assess*<br>*Not assessed*	No. of observations available		145	143	145	142	139	132	125
	Inhaled Sedation Group	Number of patients — no. (%)	*1 (1)*<br>*1 (1)*<br>*42 (60)*<br>*26 (37)*	*3 (4)*<br>*4 (6)*<br>*37 (51)*<br>*28 (39)*	*5 (7)*<br>*4 (5)*<br>*35 (48)*<br>*29 (40)*	*5 (7)*<br>*6 (8)*<br>*30 (42)*<br>*30 (42)*	*3 (4)*<br>*5 (7)*<br>*30 (43)*<br>*32 (46)*	*4 (6)*<br>*5 (7)*<br>*30 (45)*<br>*28 (42)*	*3 (5)*<br>*4 (6)*<br>*29 (45)*<br>*28 (44)*
	Intravenous Sedation Group	Number of patients — no. (%)	*0 (0)*<br>*2 (3)*<br>*10 (13)*<br>*63 (84)*	*0 (0)*<br>*1 (1)*<br>*9 (13)*<br>*61 (86)*	*0 (0)*<br>*1 (1)*<br>*7 (10)*<br>*64 (89)*	*0 (0)*<br>*4 (6)*<br>*3 (4)*<br>*64 (90)*	*3 (4)*<br>*2 (3)*<br>*5 (7)*<br>*59 (86)*	*1 (2)*<br>*3 (5)*<br>*5 (8)*<br>*56 (86)*	*3 (5)*<br>*2 (3)*<br>*4 (7)*<br>*52 (85)*
	P Value*		10^{-4}	10^{-4}	10^{-4}	10^{-4}	10^{-4}	10^{-4}	10^{-4}
**ABCDEF Bundle**									
Was the patient on the vent?	No. of observations available		183	179	176	175	171	164	154

	Inhaled Sedation Group	Number of patients — no. (%)	87 (86)	86 (88)	84 (86)	83 (86)	80 (84)	75 (82)	69 (79)
	Intravenous Sedation Group	Number of patients — no. (%)	63 (77)	63 (78)	61 (78)	58 (74)	55 (72)	54 (74)	49 (73)
	P Value*		0.10	0.08	0.19	0.06	0.06	0.19	0.37
Did the patient get an SAT?	No. of observations available		182	177	196	167	163	154	148
Did the patient get an SAT?	Inhaled Sedation Group	Number of patients — no. (%)	15 (15)	11 (11)	19 (20)	18 (19)	16 (17)	23 (27)	15 (18)
If not, reason: *Active seizures*			*0*	*0*	*0*	*0*	*0*	*0*	*1*
Alcohol withdrawal			*0*	*0*	*0*	*0*	*0*	*0*	*0*
Agitation			*0*	*0*	*0*	*1*	*0*	*0*	*0*
Paralytics/targeted deep sedation			*47*	*49*	*44*	*12*	*41*	*38*	*35*
Hemodynamic instability			*3*	*2*	*1*	*3*	*5*	*2*	*2*
Myocardial ischemia			*0*	*0*	*0*	*1*	*1*	*0*	*0*
High intracranial pressure			*0*	*0*	*0*	*0*	*0*	*0*	*0*
Respiratory instability			*32*	*25*	*22*	*25*	*22*	*19*	*17*
Patient off unit			*0*	*0*	*0*	*0*	*0*	*0*	*0*
Other			*17*	*22*	*19*	*16*	*20*	*14*	*20*
Did the patient get an SAT?	Intravenous Sedation Group	Number of patients — no. (%)	3 (4)	9 (11)	11 (15)	16 (22)	12 (17)	12 (18)	15 (23)
If not, reason: *Active seizures*			*0*	*0*	*0*	*0*	*0*	*0*	*0*
Alcohol withdrawal			*0*	*0*	*0*	*0*	*0*	*0*	*0*
Agitation			*0*	*1*	*0*	*0*	*0*	*0*	*0*
Paralytics/targeted deep sedation			*53*	*50*	*44*	*39*	*40*	*37*	*32*
Hemodynamic instability			*3*	*3*	*3*	*4*	*3*	*1*	*1*
Myocardial ischemia			*0*	*0*	*0*	*0*	*0*	*0*	*1*
High intracranial pressure			*0*	*0*	*0*	*0*	*0*	*0*	*0*
Respiratory instability			*52*	*41*	*36*	*28*	*30*	*31*	*27*
Patient off unit			*0*	*0*	*0*	*0*	*0*	*0*	*0*
Other			*5*	*5*	*6*	*5*	*6*	*7*	*6*
	P Value*		0.01	0.99	0.37	0.66	0.90	0.18	0.45
Did the patient get an SBT?	No. of observations available		180	177	172	166	163	155	148
Did the patient get an SBT?	Inhaled Sedation Group	Number of patients — no. (%)	15 (15)	15 (15)	18 (19)	21 (22)	21 (23)	27 (31)	16 (19)
If not, reason: *Did not pass SAT, no SBT*			*37*	*37*	*33*	*32*	*31*	*24*	*25*
Patient off unit			*0*	*0*	*0*	*0*	*0*	*0*	*0*
Agitation			*1*	*1*	*0*	*1*	*1*	*0*	*0*
Oxygen saturation <88%			*3*	*0*	*2*	*3*	*4*	*4*	*3*
FiO_2 >50%			*21*	*15*	*9*	*18*	*15*	*19*	*14*
PEEP >7.5 cmH_2O			*40*	*36*	*35*	*31*	*32*	*29*	*30*
Myocardial ischemia			*0*	*0*	*0*	*0*	*0*	*0*	*0*
Significant vasopressor use			*3*	*2*	*1*	*3*	*5*	*4*	*2*
Other			*15*	*20*	*17*	*14*	*13*	*8*	*16*
Did the patient get an SBT?	Intravenous Sedation Group	Number of patients — no. (%)	3 (4)	14 (18)	14 (19)	18 (25)	19 (27)	12 (18)	18 (28)
If not, reason: *Did not pass SAT, no SBT*			*48*	*38*	*39*	*33*	*30*	*32*	*27*
Patient off unit			*0*	*0*	*0*	*0*	*0*	*0*	*0*
Agitation			*0*	*1*	*0*	*0*	*0*	*1*	*0*
Oxygen saturation <88%			*0*	*0*	*2*	*0*	*1*	*1*	*1*
FiO_2 >50%			*18*	*10*	*8*	*9*	*6*	*5*	*4*
PEEP >7.5 cmH_2O			*38*	*34*	*31*	*27*	*25*	*23*	*19*
Myocardial ischemia			*0*	*0*	*0*	*0*	*0*	*0*	*0*
Significant vasopressor use			*4*	*3*	*2*	*3*	*2*	*1*	*1*
Other			*6*	*5*	*4*	*5*	*7*	*7*	*6*
	P Value*		0.01	0.72	0.99	0.69	0.56	0.06	0.23
Patient off sedation at the time of SBT	No. of observations available		18	29	32	39	40	38	34
Patient off sedation at the time of SBT	Inhaled Sedation Group	Number of patients — no. (%)	9 (60)	7 (47)	11 (61)	13 (62)	11 (53)	17 (65)	7 (44)
Patient off sedation at the time of SBT	Intravenous Sedation Group	Number of patients — no. (%)	2 (67)	6 (43)	7 (50)	8 (44)	6 (32)	7 (58)	6 (33)
	P Value*		0.83	0.84	0.53	0.28	0.18	0.68	0.53
Pain assessment documented	No. of observations available		182	182	178	176	173	165	157
Pain assessment documented	Inhaled Sedation Group	Number of patients — no. (%)	52 (51)	51 (51)	52 (52)	48 (49)	51 (53)	49 (53)	46 (52)

	Intravenous Sedation Group	Number of patients — no. (%)	39 (48)	43 (52)	43 (55)	45 (57)	44 (58)	41 (56)	35 (51)
	P Value*		0.66	0.85	0.68	0.23	0.49	0.71	0.98
Agitation-sedation scale target	No. of observations available		184	178	176	175	170	164	155
	Inhaled Sedation Group	Number of patients — no. (%)	55 (54)	55 (56)	54 (55)	51 (53)	51 (54)	51 (55)	49 (56)
	Intravenous Sedation Group	Number of patients — no. (%)	32 (39)	32 (41)	29 (38)	29 (37)	30 (40)	26 (36)	25 (37)
	P Value*		0.02	0.05	0.03	0.13	0.08	0.01	0.02
Agitation-sedation scale documented	No. of observations available		184	181	178	177	172	164	157
	Inhaled Sedation Group	Number of patients — no. (%)	92 (91)	92 (92)	92 (92)	90 (92)	89 (92)	84 (92)	81 (91)
	Intravenous Sedation Group	Number of patients — no. (%)	63 (76)	66 (81)	63 (81)	65 (82)	62 (83)	61 (84)	56 (82)
	P Value*		0.01	0.04	0.03	0.06	0.07	0.08	0.11
CAM-ICU documented	No. of observations available		170	167	165	164	160	152	142
	Inhaled Sedation Group	Number of patients — no. (%)	27 (30)	27 (31)	26 (30)	25 (29)	25 (29)	23 (28)	24 (31)
	Intravenous Sedation Group	Number of patients — no. (%)	6 (7)	4 (5)	4 (5)	6 (8)	7 (9)	4 (6)	5 (8)
	P Value*		10^{-4}	10^{-4}	10^{-4}	0.001	0.002	10^{-4}	0.001
Mobility safety screen passed<br>*If not, reason*<br>*RASS -4/-5*<br>*FiO2 >60%*<br>*PEEP >10 cmH2O*<br>*Myocardial ischemia (within 24h)*<br>*Arrhythmia (within 24h)*<br>*Increasing needs for vasopressors in last 2h*	No. of observations available		167	161	158	157	153	149	138
	Inhaled Sedation Group	Number of patients — no. (%)	12 (14)	10 (12)	12 (15)	13 (16)	16 (21)	18 (23)	20 (27)
			65	*59*	*55*	*53*	*54*	*49*	*43*
			7	*4*	*3*	*3*	*4*	*7*	*4*
			19	*17*	*17*	*16*	*16*	*13*	*9*
			0	*0*	*0*	*1*	*1*	*0*	*0*
			1	*0*	*0*	*1*	*1*	*0*	*1*
			1	*0*	*1*	*1*	*1*	*1*	*1*
	Intravenous Sedation Group	Number of patients — no. (%)	8 (10)	14 (18)	15 (19)	19 (24)	16 (21)	17 (24)	16 (25)
			60	*55*	*50*	*43*	*43*	*41*	*33*
			4	*3*	*4*	*6*	*3*	*4*	*3*
			26	*18*	*19*	*20*	*15*	*13*	*12*
			0	*0*	*0*	*0*	*0*	*0*	*0*
			0	*1*	*0*	*0*	*1*	*1*	*1*
			3	*2*	*2*	*2*	*1*	*1*	*2*
	P Value*		0.42	0.36	0.48	0.22	0.90	0.97	0.71
Exercise or mobilization this day<br>*If not, reason*<br>*Effect of sedative administration*<br>*No staff available*<br>*Patient/family refused*<br>*Patient off unit*<br>*Clinical team refused*<br>*Unknown*<br>*Other*	No. of observations available		172	169	167	165	162	156	146
	Inhaled Sedation Group	Number of patients — no. (%)	20 (22)	23 (26)	22 (25)	22 (25)	25 (29)	24 (25)	23 (29)
			60	*57*	*53*	*53*	*51*	*47*	*43*
			6	*6*	*7*	*6*	*5*	*7*	*6*
			0	*0*	*0*	*0*	*0*	*0*	*0*
			0	*0*	*0*	*0*	*0*	*0*	*0*
			0	*0*	*1*	*0*	*0*	*1*	*1*
			1	*1*	*2*	*3*	*2*	*2*	*2*
			5	*3*	*4*	*2*	*3*	*3*	*4*
	Intravenous Sedation Group	Number of patients — no. (%)	6 (7)	9 (11.25)	9 (12)	14 (18)	13 (17)	13 (18)	12 (18)
			59	*55*	*54*	*47*	*48*	*47*	*43*
			5	*5*	*5*	*6*	*6*	*4*	*3*
			0	*0*	*0*	*0*	*0*	*0*	*0*
			0	*0*	*0*	*0*	*0*	*0*	*0*
			0	*0*	*0*	*1*	*0*	*0*	*0*
			1	2	*2*	*4*	2	*1*	2
			4	*2*	*3*	*0*	0	*1*	1
	P Value*		0.01	0.02	0.03	0.25	0.09	0.12	0.14
	No. of observations available		18	20	21	24	24	24	23

What was the highest level of mobilization performed?	Inhaled Sedation Group	Number of patients — no. (%)	*12 (92)*	*11 (85)*	*10 (77)*	*9 (75)*	*10 (17)*	*9 (64)*	*8 (62)*
Level 1: Active range of motion			*0 (0)*	*0 (0)*	*1 (8)*	*1 (8)*	*2 (14)*	*1 (7)*	*1 (8)*
Level 2: Dangle			*1 (8)*	*2 (15)*	*2 (15)*	*2 (17)*	*2 (14)*	*4 (29)*	*4 (31)*
Level 3: Active transfers			*0 (0)*	*0 (0)*	*0 (0)*	*0 (0)*	*0 (0)*	*0 (0)*	*0 (0)*
Level 4: March/walk	Intravenous Sedation Group	Number of patients — no. (%)	*5 (100)*	*6 (86)*	*5 (63)*	*8 (67)*	*7 (70)*	*6 (60)*	*8 (62)*
			0 (0)	*1 (14)*	*1 (13)*	*0 (0)*	*0 (0)*	*1 (10)*	*0 (0)*
			0 (0)	*0 (0)*	*2 (25)*	*3 (25)*	*3 (30)*	*3 (30)*	*2 (20)*
			0 (0)	*0 (0)*	*0 (0)*	*1 (8)*	*0 (0)*	*0 (0)*	*0 (0)*
	P Value*		0.52	0.23	0.78	0.52	0.35	0.96	0.52
Visit from family members or proxies	No. of observations available		182	178	176	173	168	160	152
Yes	Inhaled Sedation Group	Number of patients — no. (%)	*5 (5)*	*3 (3)*	*3 (3)*	*5 (5)*	*4 (4)*	*3 (3)*	*2 (2)*
No			*76 (76)*	*76 (78)*	*74 (76)*	*70 (74)*	*71 (76)*	*69 (78)*	*64 (75)*
Unknown			*19 (19)*	*18 (19)*	*20 (21)*	*20 (21)*	*18 (19)*	*16 (18)*	*19 (22)*
	Intravenous Sedation Group	Number of patients — no. (%)	*2 (2)*	*3 (4)*	*2 (3)*	*3 (4)*	*1 (1)*	*5 (7)*	*2 (3)*
			49 (60)	*46 (57)*	*42 (53)*	*44 (56)*	*41 (55)*	*37 (51)*	*35 (52)*
			31 (38)	*32 (40)*	*35 (44)*	*31 (40)*	*33 (44)*	*30 (42)*	*30 (45)*
	P Value*		0.02	0.01	0.003	0.03	0.002	0.001	0.01

* *P* values were calculated for comparisons between patients receiving inhaled sedation (for at least 24 hours) and those receiving only intravenous sedation through day 28. Percentages are expressed out of available individuals for each variable, and may not sum to 100% due to rounding. Quantitative variables are expressed as median [IQR], qualitative variables as number (%), unless specified otherwise. SAT: spontaneous awakening trial; SBT: spontaneous breathing trial. RASS: Richmond agitation and sedation scale; BPS: behavioral pain scale; BIS: bispectral index; CAM-ICU: confusion assessment method for the intensive care unit; FiO_2: inspired fraction of oxygen; PEEP: positive end expiratory pressure.

References

1. von Elm E, Altman DG, Egger M, Pocock SJ, Gøtzsche PC, Vandenbroucke JP, et al. The Strengthening the Reporting of Observational Studies in Epidemiology (STROBE) statement: guidelines for reporting observational studies. J Clin Epidemiol. 2008 Apr;61(4):344–9.
2. Harris PA, Taylor R, Thielke R, Payne J, Gonzalez N, Conde JG. Research electronic data capture (REDCap)--a metadata-driven methodology and workflow process for providing translational research informatics support. J Biomed Inform. 2009 Apr;42(2):377–81.
3. Devlin JW, Skrobik Y, Gélinas C, Needham DM, Slooter AJC, Pandharipande PP, et al. Clinical Practice Guidelines for the Prevention and Management of Pain, Agitation/Sedation, Delirium, Immobility, and Sleep Disruption in Adult Patients in the ICU. Crit Care Med. 2018 Sep;46(9):e825–73.
4. Bunn, A.; Korpela, M. An Introduction to dplR. Processed with dplR 1.7.2 in R version 4.0.3. 2013; Available from: http://r.meteo.uni.wroc.pl/web/packages/dplR/vignettes/intro-dplR.pdf
5. Jabaudon M, Boucher P, Imhoff E, Chabanne R, Faure JS, Roszyk L, et al. Sevoflurane for Sedation in Acute Respiratory Distress Syndrome. A Randomized Controlled Pilot Study. Am J Respir Crit Care Med. 2017 Mar 15;195(6):792–800.
